# TRAF inhibition drives cancer cell apoptosis and improves retinoic acid sensitivity in multiple cancers models

**DOI:** 10.1007/s12672-023-00703-5

**Published:** 2023-06-30

**Authors:** Kun Zhong, Xiaojun Liu, Weihua Ding, Lizhong Peng, Xuhui Zeng, Yayun Gu

**Affiliations:** Medical School, Nantong University, 19 Qixiu Road, Nantong, 226001 Jiangsu Province People’s Republic of China

**Keywords:** TRAF inhibition, Retinoic acid sensitivity, cancer cell apoptosis

## Abstract

TNF receptor-associated factors (TRAFs) are signaling adaptor proteins that play a crucial role in regulating cellular receptors’ signaling transduction to downstream pathways and exert multifaceted roles in regulating signaling pathways, cell survival, and carcinogenesis. The 13-cis-retinoic acid (RA), an active metabolite of vitamin A, exhibits anti-cancer properties, but the development of retinoic acid resistance poses a challenge in clinical application. This study aimed to investigate the relationship between TRAFs and retinoic acid sensitivity in various cancers. Here, we revealed that TRAFs’ expression varied significantly across The Cancer Genome Atlas (TCGA) cancer cohorts and human cancer cell lines. Additionally, inhibiting TRAF4, TRAF5, or TRAF6 improved retinoic acid sensitivity and reduced colony formation in ovarian cancer and melanoma cells. Mechanistically, knocking down TRAF4, TRAF5, or TRAF6 in retinoic acid-treated cancer cell lines increased the levels of procaspase 9 and induced cell apoptosis. Further in vivo studies using the SK-OV-3 and MeWo xenograft models confirmed the anti-tumor effects of TRAF knockdown combined with retinoic acid treatment. These findings support that combination therapy with retinoic acid and TRAF silencing may offer significant therapeutic advantages in treating melanoma and ovarian cancers.

**Supplementary Information:**

The online version contains supplementary material available at 10.1007/s12672-023-00703-5.

## Introduction

The tumor necrosis factor receptor-associated factor (TRAF) family (TRAF1-TRAF7) is involved in regulating numerous cellular processes such as embryonic development [1], tissue homeostasis [2], inflammatory responses [3–5], and carcinogenesis [6]. All TRAF members, except for TRAF7, contain a typical C-terminal homology motif known as the TRAF domain, which can couple to other TRAF proteins as well as the cytoplasmic domain of various cellular receptors [7–8]. TRAFs act as intracellular signal transducers and E3 ubiquitin ligases [9], and are involved in regulating downstream signaling pathways of various cellular receptors, including the TNF receptor superfamily (TNF-R), Toll-like receptors (TLRs), retinoic acid-inducible gene-I receptor (RLRs), NOD-like receptors (NLRs), and cytokine receptors [10–14].

Various human cancers display genetic alterations and overexpression of TRAF proteins [6]. B-cell neoplasms and human carcinomas such as melanoma, ovarian cancer, glioblastoma, and breast cancer have been found to implicate TRAF1, TRAF2, TRAF3, and TRAF5 [15–20]. While TRAF2 and TRAF3 have oncogenic and tumor-suppressive activities [17, 19], TRAF1 and TRAF5 function as oncogenes [15, 20]. Specifically, TRAF2 activates the NF-kappa-B pathway, triggering the transcription of cytoprotective and antiapoptotic genes [17]. TRAF5 promotes NF-kappa-B activation and regulates cell survival and apoptosis [20]. TRAF4 and TRAF6, noncanonical members of the TRAF family, are widely recognized as oncogenes in various human malignancies, including lung cancer, breast cancer, ovarian cancer, melanomas, neurogenic tumors, colon cancer, osteosarcoma, and pancreatic cancer [21–25]. Recently, TRAF7, a newly discovered member of the TRAF family, has been found to be involved in the development of several human malignancies, bringing it into the spotlight as a potential tumor suppressor gene [26]. Given the role of TRAFs in cell signaling regulation and tumorigenesis, targeting TRAFs and TRAF-dependent signaling pathways could be crucial for enhancing the efficacy of current human cancer therapy methods [27].

Retinoic acid, a metabolite of vitamin A, is a widely recognized anticancer drug that has the ability to control cell growth and differentiation, as well as induce cell death. It functions as an inducible transcription factor that interacts with retinoic acid receptors (RARs) or retinoid X receptors (RXRs) to regulate the expression of retinoid-responsive specific genes [28]. Retinoic acid has shown promise in treating various types of cancer, but resistance to the therapy has been reported in solid tumors [29–31]. Therefore, identifying the molecular biomarkers that regulate retinoic acid sensitivity could be crucial for enhancing the efficacy of retinoid therapy in the relevant cancer types.

Herein, we explored the potential correlation between the TRAF family and retinoic acid sensitivity in several human carcinomas. Our findings indicate that inhibition of TRAFs enhances the sensitivity of multiple cancer types to retinoic acid therapy by promoting cell apoptosis. We identified the TRAF family as a potential biomarker for predicting retinoic acid resistance and a potential target for improving retinoid therapy.

## Materials and methods

### Reagents and cell culture

The cell lines used in the study were purchased from American Type Culture Collection (ATCC, Manassas, VA, USA) and the European Collection of Authenticated Cell Cultures (ECACC, Salisbury, UK): human melanoma cell line MeWo (ATCC HTB-65), human ovarian cancer cell line SK-OV-3 (ATCC HTB-77), human malignant glioblastoma cell line U251 (ECACC 09063001), human prostate cancer cell line 22RV1(ATCC CRL-2505), human breast cancer cell line BT549 (ATCC HTB-122). MeWo, SK-OV-3 and U251 were cultured in Dulbecco’s modified Eagle medium (DMEM, Gibco, USA), 22RV1 and BT549 were maintained in Roswell Park Memorial Institute 1640 medium (RPMI, Gibco, USA). All media were supplemented with 10% fetal bovine serum (35-010-CV, fetal bovine serum, Corning, USA) and 1% penicillin/streptomycin (15140-122, Gibco, USA), and cells were maintained at 37 °C with 5% CO_2_.

The 13-*cis*-Retinoic acid (≥ 98% HPLC, R3255) was purchased from Sigma. Antibodies used for immunoblotting and shRNA Lentiviral Particles were listed in Supplementary Table 1. Scrambled siRNA control and TRAF siRNAs targeting TRAF1, TRAF2, TRAF3, TRAF4, TRAF5, TRAF6, and TRAF7 were purchased from Horizon Discovery (Waterbeach, UK). The sequences of shRNA and siRNA were listed in Supplementary Table 2.

### siRNA transfection

Lipofectamine RNAiMAX Reagent (13,778,150, Thermo Fisher Scientific, Waltham, MA, USA) was used to transfect siRNAs. In brief, cells were plated into 60 mm dishes at a destiny of 8 × 10^5^ cells per dish and cultured overnight. Then 8 nM siRNA were gently mixed with 16 µL RNAiMAX reagent in 100 µL Opti-MEM and incubated for 10 min, and then the mixture was added to the cells. The transfected cells were then subjected to cell cytotoxicity assays.

### Lentiviral particles transduction

Prior to lentivitus transduction, cells were plated in a 6-well plate (5 × 10^4^ cells per well) and cultured overnight. Then 1 × 10^6^ infectious units (IFUs) of lentivirus and polybrene (sc-134,220, Santa Cruz, CA, USA) at the final concentration of 8 µg/mL were gently added to the cells. Cells were cultured for an additional 72 h before 2 µg/mL puromycin (sc-108,071, Santa Cruz, CA, USA) was used to select positive knockdown cells.

### CyQUANT direct cell proliferation assay

Cytotoxicity to human cancer cell lines was determined using the CyQUANT direct cell proliferation assay kit (C35012, Thermo Fisher Scientific, Waltham, MA, USA). Cells were seeded into 96-well plates at a density of 2000–4000 cells/well. After overnight incubation, cells were treated with different concentrations of retinoic acid (0.125, 0.25, 0.5, 1, 2, 4, 8, 16, and 32 µM) for 5 days. Then, 10 µL of fresh CyQUANT mixture was prepared and added to each well. Plates were then incubated at 37 °C for 1 h before being scanned by an Infinite M1000 Pro plate reader with 480 nm excitation and 520 nm emission filters (Tecan AG, Switzerland).

### Colony formation assay

The colony formation assay was carried out as previously described. SK-OV-3 cells/ MeWo cells (shNEG and shTRAF cells) were seeded at a density of 1.0 × 10^3^ cells per well into 6 well plates and cultured in 8 µM retinoic acid for 2 weeks in a humidified atmosphere with 5% CO_2_ at 37 °C. The formed colonies were fixed with ethanol, washed twice with pre-cold PBS, and then stained overnight with 0.5% w/v crystal violet before being counted using the ChemiDoc™ Touch Imaging System (Bio-Rad, Hercules, CA, USA). Three independent repeating tests were performed for all samples.

### Western blotting

To extract protein from the cell samples, NETN lysis buffer (20 mM Tris HCl, 0.5 mM EDTA, 100 mM NaCl, NP-40) supplemented with Protease inhibitor cocktail (11,836,153,001, Roche, Basel, Switzerland) and phosphatase inhibitor (4906845001, Roche, Basel, Switzerland) was added to cells. The cells were lysed on ice for 30 min in cold NETN buffer, followed by being sonicated and centrifuged at 12,000 rpm for 15 min at 4 °C. The supernatant was diluted with SDS loading buffer and heated at 95 °C for 8 min. The protein concentration was measured using a Quick Start™ Bradford assay (5000205, Bio-Rad, Hercules, CA, USA) according to the manufacturer’s instructions. Totally 20 µg protein from each sample was loaded in 4–20% TGX SDS gels (Bio-Rad, Hercules, CA, USA) and transferred to polyvinylidene difluoride (PVDF) membranes. The membranes were blocked for 1 h at room temperature in 5% nonfat milk dissolved in 1×TBST before being incubated overnight with primary antibodies (Supplementary Table 1) at 4 °C. After being washed with 1×TBST, the membranes were incubated with secondary antibodies for 1 h at room temperature. All blots were developed with Supersignal West Pico chemiluminescent ECL kit (Thermo Fisher Scientific, Waltham, MA, USA) and imaged by a ChemiDoc™ Touch Imaging System (Bio-Rad, Hercules, CA, USA).

### Quantitative PCR

Quick RNA MiniPrep kit (R1055, ZYMO RESEARCH, Irvine, CA, USA) was used to isolate total RNA from indicated cells according to the manufacturer’s instructions. The Purity and concentration of total RNAs were determined using a Nanodrop spectrophotometer with 260/280 ratios of ~ 2.0. Amplification reactions were conducted using the Power SYBR™ Green RNA-to-CT™ 1-Step Kit (4391178, Thermo Fisher Scientific) with a 7500 Realtime PCR device (Applied Biosystems, California, USA). The specific primer sequences are listed in Supplementary Table 3. All the experiments were performed in triplicate with GAPDH as an internal control. The relative mRNA expression levels were compared using the 2^−ΔΔCt^ method.

### Animal study

Six-week-old female athymic nude Foxn1^nu^ mice from the Biomedical Research Institute of Nanjing University were used to establish SK-OV-3 and MeWo tumors. 1 × 10^7^ SK-OV-3 or MeWo cells suspended in 100µL PBS were implanted subcutaneously per mouse. Once the average tumor diameters for all groups reached 6 mm, mice were subjected to siNEG (siNegtive), siNEG + retinoic acid, siTRAF, or siTRAF + retinoic acid treatment. For the injection, 0.2 µg/µL siRNAs mixed with invivofectamine 3.0 reagent solution were administered intratumorally per tumor. Retinoic acid (25 mg/kg) suspended in corn oil was given by gavage feeding for each dose. Tumor sizes were measured by a caliper and tumor volumes were calculated using the following formula: Tumor volumes = length × width^2^/2.

All animal experiments were approved by Nantong University Institutional Animal Care and Use Committee (IACUC) and were executed under the guidelines of the Nantong University IACUC. Following the guidelines for the ethical review of laboratory animal welfare People’s Republic of China National Standard GB/T 35892−2018 [32], the tumor burden should not exceed 2000 mm^3^. The IACUC protocol in this study follows the guidelines, and mice carrying tumors larger than 2000 mm^3^ were euthanized in accordance with the IACUC protocol.

### TRAF mRNA expression analysis

The cancer dataset in The Cancer Genome Atlas (TCGA) was downloaded to retrieve data on TRAF mRNA expression in human samples. Analyses of TRAF mRNA levels were performed using the tool provided by UCSC XENA (http://xena.ucsc.edu/).

### Statistics

The statistical data were presented as mean ± standard deviation (SD) of 3 independent experiments. All statistical tests were performed using GraphPad Prism software. A two-tailed unpaired Student’s *t* test was utilized to determine the statistical significance for two group comparisons. One-way ANOVA or two-way ANOVA along with Bonferroni’s multiple comparisons were used when multiple variables were compared. IC50 was calculated using Nonlinear regression (Dose-response-Inhibition). Differences in animal survival distributions between treatment groups were compared using the log-rank test. P < 0.05 was considered statistically significant.

## Results

### TRAFs were differentially expressed in human tumor samples

**Fig. 1:**
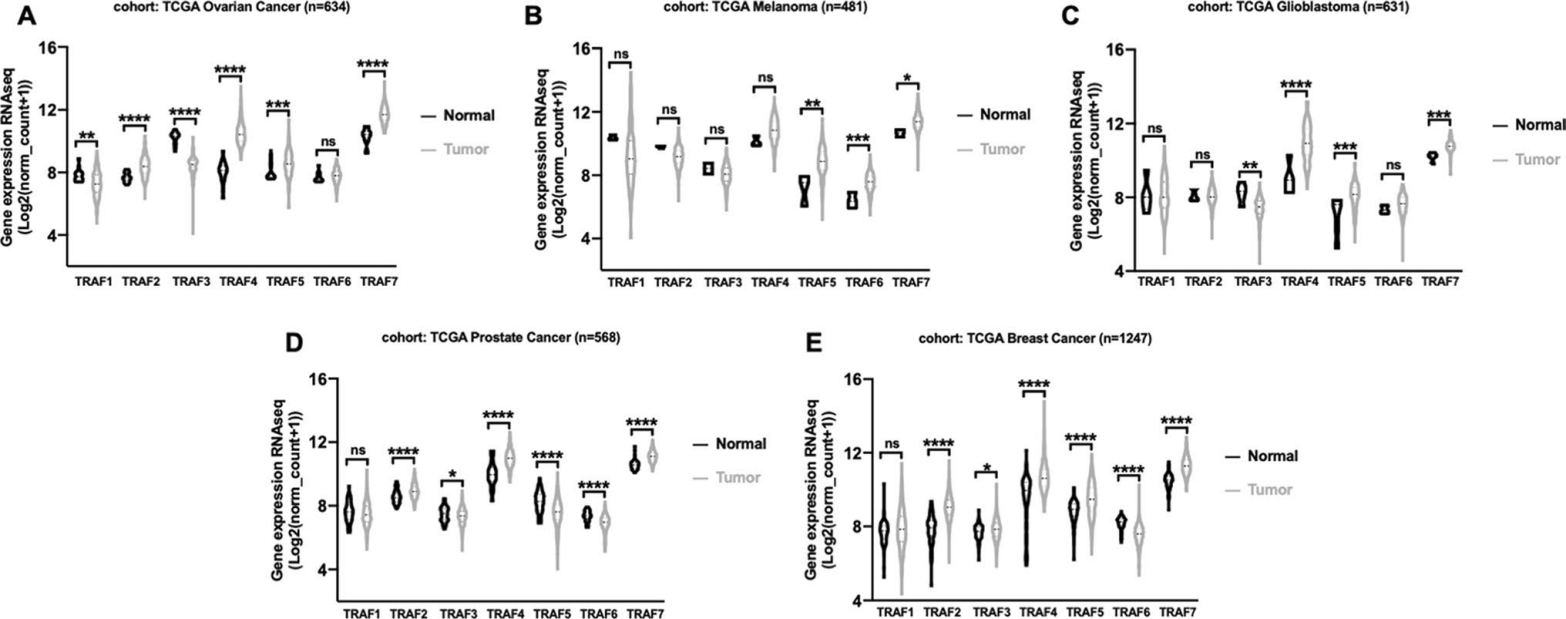
Gene expression of TRAF family in various human malignancies. The relative mRNA levels of TRAF family members (TRAF1-TRAF7) in cancerous and normal tissues were compared in five different cohorts: **A** ovarian cancer, **B** TCGA melanoma, **C** TCGA glioblastoma, **D** TCGA prostate cancer, and (E) TCGA breast cancer. The mRNA expression data were extracted from the TCGA database and analyzed by two-tailed unpaired Student’s *t*-test. ns, not significant. P < 0.05 was considered statistically significant. (*P < 0.05; **P < 0.01; ***P < 0.001; ****P < 0.0001

To clarify the potential association between the TRAF family (TRAF1-TRAF7) and human cancers, we investigated the TRAFs RNA expression data extracted from the TCGA database. Data from five cohorts, including an ovarian cancer cohort (n = 634), a melanoma cohort (n = 481), a glioblastoma cohort (n = 631), a prostate cancer cohort (n = 568), and a breast cancer cohort (n = 1247), were analyzed. As shown in Fig. 1A and E, TRAF1- TRAF7 were shown to be differentially expressed in various human cancers, and upregulation of TRAF expression was observed in tumor tissues compared to normal tissues in multiple cancer cohorts. Specifically, TRAF7 exhibited the highest average mRNA level among the TRAF family, followed by TRAF4, while TRAF6 showed the lowest average mRNA level according to the TCGA cancer database.

**Fig. 2:**
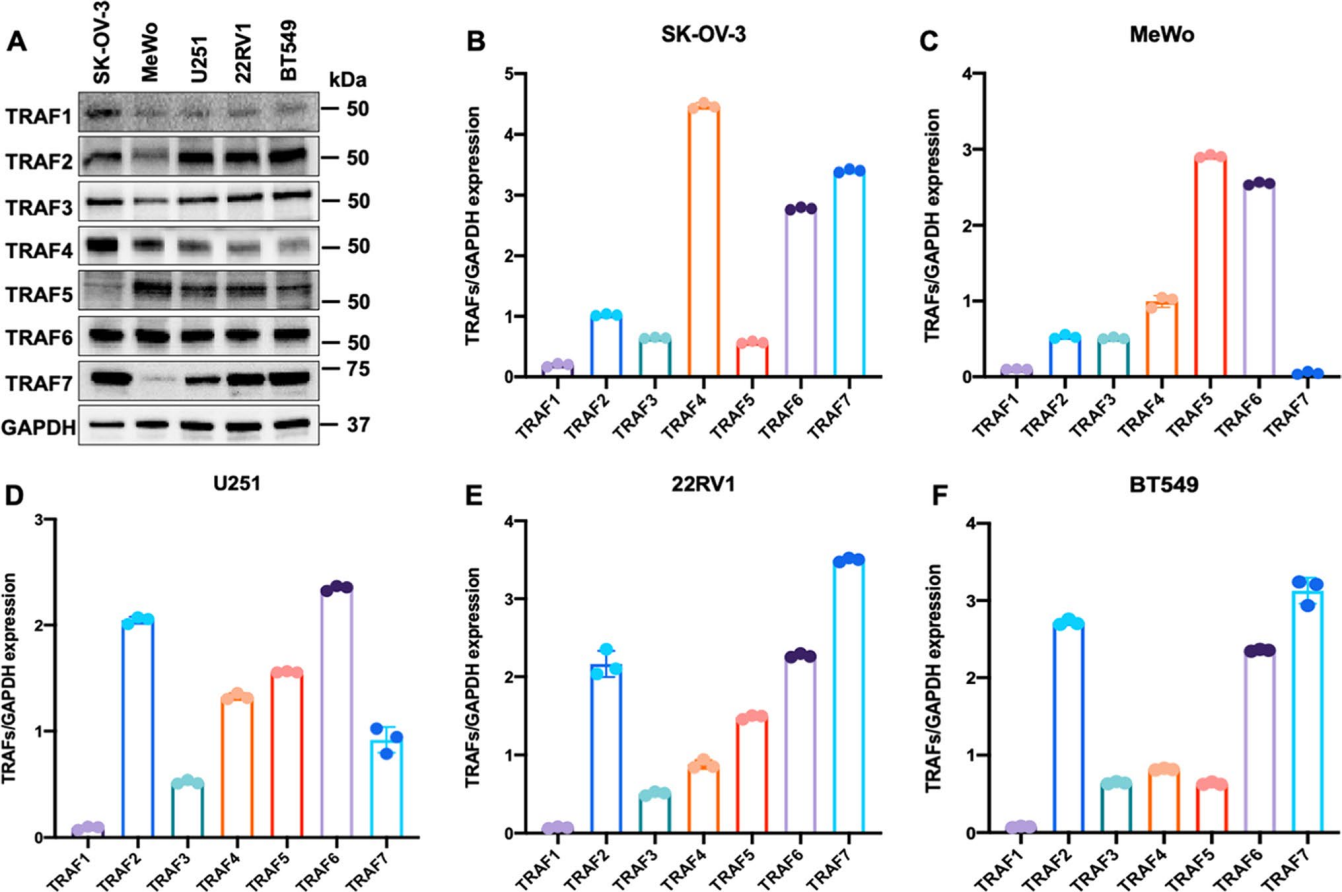
Protein levels of TRAF family (TRAF1-TRAF7) in human cancer cell lines. **A** Protein expression levels of TRAFs in ovarian cancer cell SK-OV-3, human melanoma cell MeWo, human glioblastoma cell U251, human prostate cancer cell 22RV1, and human breast cancer cell BT549. **B–F** Relative protein levels of TRAFs in detected cell lines

Next, we evaluated the protein levels of TRAF1-TRAF7 in different human cancer cell lines, including the human ovarian cancer cell SK-OV-3, human melanoma cell MeWo, human malignant glioblastoma cell U251, human prostate cancer cell 22RV1, and human breast cancer cell BT549. Our results revealed that TRAF1 and TRAF4 were highly expressed in SK-OV-3 cells but not in the other cell lines. TRAF2 and TRAF7 showed high protein levels in all cancer cell lines, except for the MeWo cells. On the contrary, the TRAF5 level was especially high in MeWo cells compared to the other cell lines. Furthermore, TRAF3 and TRAF6 were equivalently expressed in all the cancer cell lines detected (Fig. 2).

### **Inhibition of TRAF4, TRAF5, or TRAF6 improved retinoic acid sensitivity in human ovarian SK-OV-3 cancer cell and human melanoma MeWo cell**

**Fig. 3:**
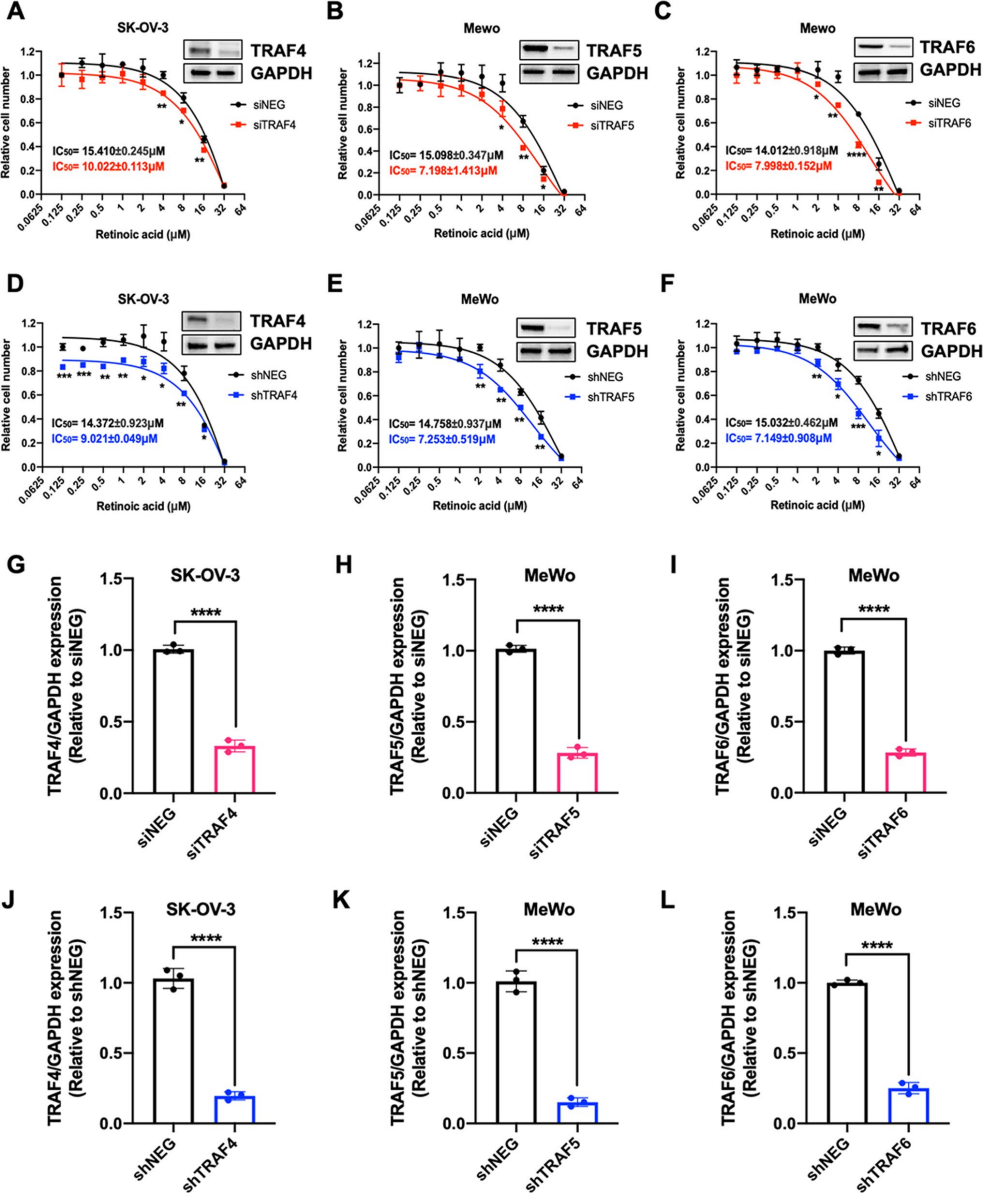
Downregulation of TRAF4, TRAF5 and TRAF6 increased retinoic acid sensitivity in human ovarian cancer and melanoma cell lines. **A**–**C** SK-OV-3 and MeWo cells transfected with siNegative control (siNEG) or siRNA targeting TRAFs (siTRAF4, siTRAF5, siTRAF6) were treated with retinoic acid at various concentrations. **D**–**F** SK-OV-3 and MeWo cells transduced with shNegative control (shNEG) or TRAFs shRNA (shTRAF4, shTRAF5, shTRAF6) lentivirus were treated with retinoic acid at various concentrations. TRAFs knockdown was confirmed by Western blot analysis. Relative cell number was and determined using the CyQUANT assay (two-tailed unpaired Student’s t test). Nonlinear regression (Dose-response-Inhibition) was used for the calculation IC_50_ of Retinoic acid. **G**–**I** TRAF4/TRAF5/TRAF6 knockdown by siRNA in SK-OV-3 or MeWo cells. **J–L** TRAF4/TRAF5/TRAF6 knockdown by shRNA in SK-OV-3 or MeWo cells. TRAF protein levels were compared with two-tailed unpaired Student’s *t *test. All values represent the means of at least three independent experiments ± SD. P < 0.05 was considered statistically significant. (^*^P < 0.05; ^**^P < 0.01; ^***^P < 0.001; ^****^P < 0.0001. *P indicates comparisons with siNEG or shNEG group.)

Previous research has demonstrated that tumor necrosis factor-related factors, such as the tumor necrosis factor-related apoptosis-inducing ligand (TRAIL), contributed to the therapeutic efficacy of retinoids [33–35]. Here, we hypothesized that the TRAF family could be crucial in regulating retinoic acid sensitivity in human cancer cell lines. We examined the impacts of TRAF knockdown on retinoic acid sensitivity in various human cancer cell lines. After transfecting SK-OV-3, MeWo, U251, 22RV1, and BT549 cells with siRNAs targeting different TRAFs, cells were treated with increasing concentrations (0.125–32 µM) of retinoic acid for 5 days. CyQUANT direct cell proliferation assays were performed to determine the relative cell numbers after the retinoic acid treatment (Fig. 3 and Additional file 1: Fig. S1–Fig. S5). Unexpectedly, siRNA-mediated knockdown of TRAF4, rather than other TRAFs, drastically reduced the IC_50_ of retinoic acid in SK-OV-3 cells (from 15.410 µM ± 0.245 µM to 10.022 µM ± 0.113 µM) (Fig. 3A). Meanwhile, both TRAF5 and TRAF6 knockdown significantly decreased the IC_50_ of retinoic acid in MeWo cells (from 15.098 µM ± 0.347 µM to 7.198 µM ± 1.413 µM for TRAF5 knockdown, from 14.012 µM ± 0.918 µM to 7.998 µM ± 0.152 µM for TRAF6 knockdown) (Fig. 3B, C). Western blot analysis confirmed the downregulated protein levels of TRAF4, TRAF5, and TRAF6 post the siRNA transfection (Fig. 3A, C, G, I). For the other TRAFs, siRNA-mediated gene knockdown showed no impact on cell proliferation under retinoic acid treatment (Additional file 1: Fig. S1, S2). Influences of TRAF family knockdown on the sensitivity to retinoic acid in U251, 22RV1, and BT549 cells were not observed (Additional file 1: Fig. S3–S5).

To further validate the impact of TRAF4, TRAF5, and TRAF6 inhibition on retinoic acid sensitivity, TRAF4, TRAF5, and TRAF6 were stably knocked down in SK-OV-3 and MeWo cells using lentiviruses expressing the TRAF targeted short hairpin RNA (shRNA) sequences, respectively (Fig. 3D, F, J–L). Accordingly, the shRNA-mediated TRAF4 inhibition decreased the IC_50_ of retinoic acid in SK-OV-3 cell (from 14.372 µM ± 0.923 µM to 9.021 µM ± 0.049 µM). Both the silencing of TRAF5 and TRAF6 by shRNAs decreased the IC_50_ of retinoic acid in MeWo cells (from 14.758 µM ± 0.937 µM to 7.253 µM ± 0.519 µM for TRAF4 inhibition and from 15.032 µM ± 0.462 µM to 7.149 µM ± 0.908 µM for TRAF6 inhibition). All results of shRNA-mediated knockdown were consistent with the findings of siRNA-mediated knockdown.

### Knockdown of TRAF4, TRAF5, or TRAF6 suppressed the colony formation of human ovarian cancer cell SK-OV-3 and human melanoma cell MeWo

**Fig. 4:**
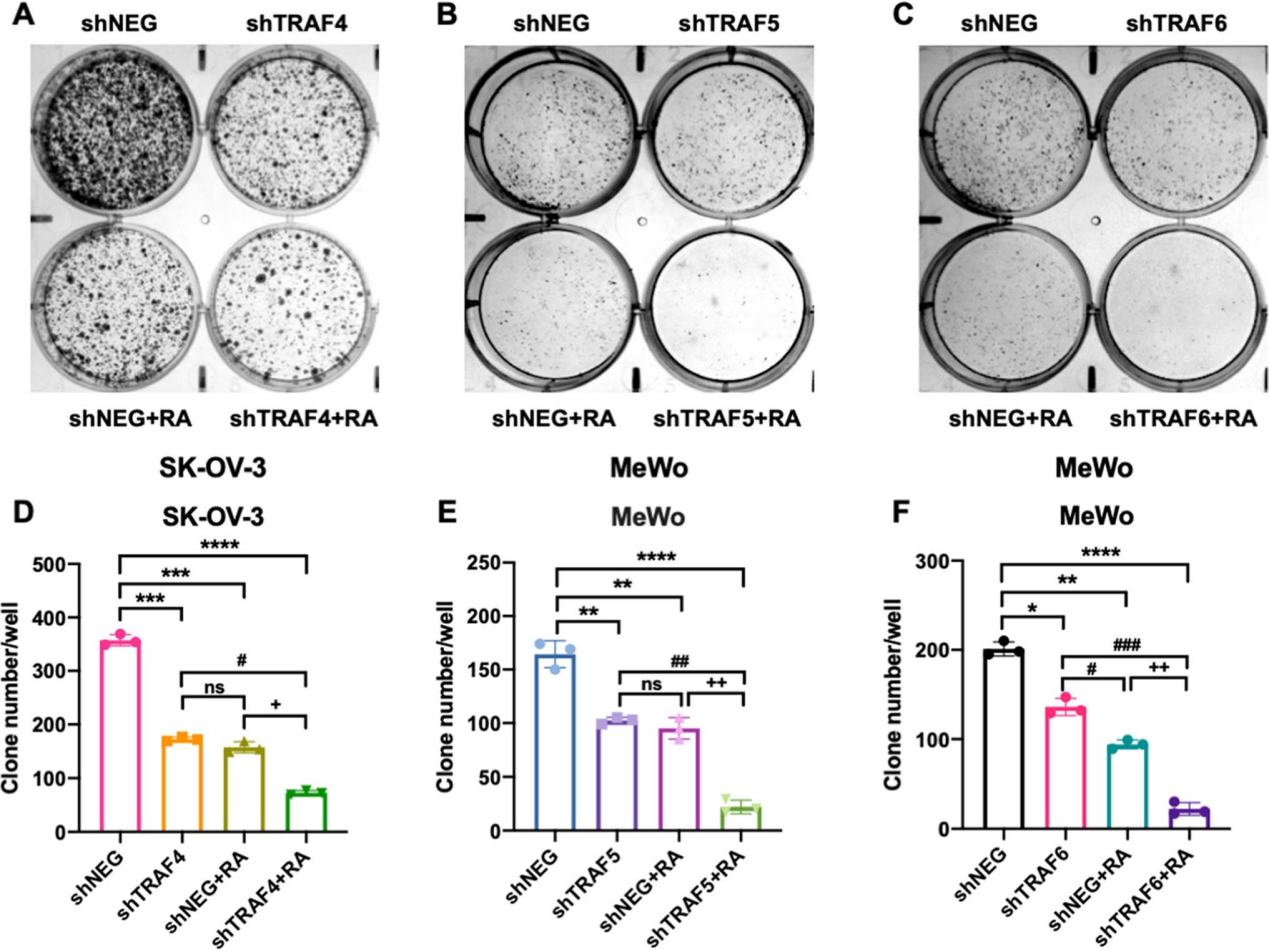
TRAF4, TRAF5, and TRAF6 knockdown inhibited colony formation of human ovarian cancer and melanoma cells. **A** Colony formation assays following shNegtive control (shNEG) or shTRAF4 lentivirus transduction, with or without 8 µM retinoic acid treatment in SK-OV-3 cells. **B**, **C** Colony formation assays following shNEG or shTRAF5 lentivirus transduction, with or without 8 µM retinoic acid treatment in MeWo cells. **D**–**F** Quantification of cell colonies from different groups. Statistical significance was calculated using a one-way analysis of variance (ANOVA) with Bonferroni’s multiple-comparison test. All values represent the means of at least three independent experiments ± SD. ns not significant, P < 0.05 was considered statistically significant. (^*^P < 0.05; ^**^P < 0.01; ^***^P < 0.001; ^****^P < 0.0001. ^#^P < 0.05; ^##^P < 0.01; ^###^P < 0.001. ^+^P < 0.05; ^++^P < 0.01. ^*^P indicates comparisons with shNEG group; ^#^P indicates comparisons with shTRAF group; ^+^P indicates comparisons with shNEG + RA group)

To further study TRAFs’ roles in human cancer regulation, we then performed clonogenic growth assays with shTRAF4 knockdown and shNegative control SK-OV-3 cells, shTRAF5 knockdown and shNegative control MeWo cells, shTRAF6 knockdown and shNegative control MeWo cells. As presented in Fig. 4A–C, shTRAF4 knockdown SK-OV-3 cells, shTRAF5 knockdown MeWo cells, and shTRAF6 knockdown MeWo cells formed fewer colonies than the control cells without the retinoic acid treatment, indicating that TRAF4, TRAF5, or TRAF6 knockdown alone has successfully suppressed the growth of human ovarian cancer or human melanoma cells. Given our findings that inhibiting TRAF4, TRAF5, or TRAF6 can improve retinoic acid sensitivity in SK-OV-3 or MeWo cells, we further tested whether a combination of TRAF inhibition plus retinoic acid treatment would be more effective in inhibiting SK-OV-3 or MeWo cell growth. Compared to the shNegative control group, the shTRAF4, TRAF5, or TRAF6 stable knockdown followed by retinoic acid treatment considerably inhibited colony formation of SK-OV-3 or MeWo cells, as revealed by the CyQUANT cell proliferation assay results.

The numbers of cell clones were quantified (Fig. 4D–F). In SK-OV-3 cells, silencing TRAF4 plus retinoic acid treatment dramatically decreased the number of clones to 20.8%, compared to 49.4% with 8 µM retinoic acid alone. In MeWo cells, TRAF5 and TRAF6 inhibition, when combined with retinoic acid, exhibited potent inhibitory effects (15.6% and 13.2%, respectively) compared to the 8 µM retinoic acid control group. Collectively, TRAF inhibition combined with retinoic acid dramatically reduced the colony formation of human ovarian cancer and melanoma cells.

### TRAF inhibition promoted the expression of pro-apoptotic genes and enhanced the sensitivity to retinoic acid in human cancer cell lines

Retinoic acid (RA) was shown to regulate cell growth via apoptosis, apoptosis-related signaling pathways. We speculated whether inhibiting TRAFs might promote retinoic acid-induced cell apoptosis in human cancer cell lines. To test our hypothesis, we investigated the expression of several molecules associated with apoptosis, such as oncoproteins (Bcl-2), the apoptosis inhibitor Survivin, proapoptotic proteins (p53 and procaspase 9), Activator protein 1 (AP-1) and Interferon regulatory factor-1 (IRF-1), in the shRNA transduced cells with or without retinoic acid co-treatment. Western blot and RT-qPCR analyses were employed to evaluate protein and mRNA levels of all the aforementioned proteins.

**Fig. 5:**
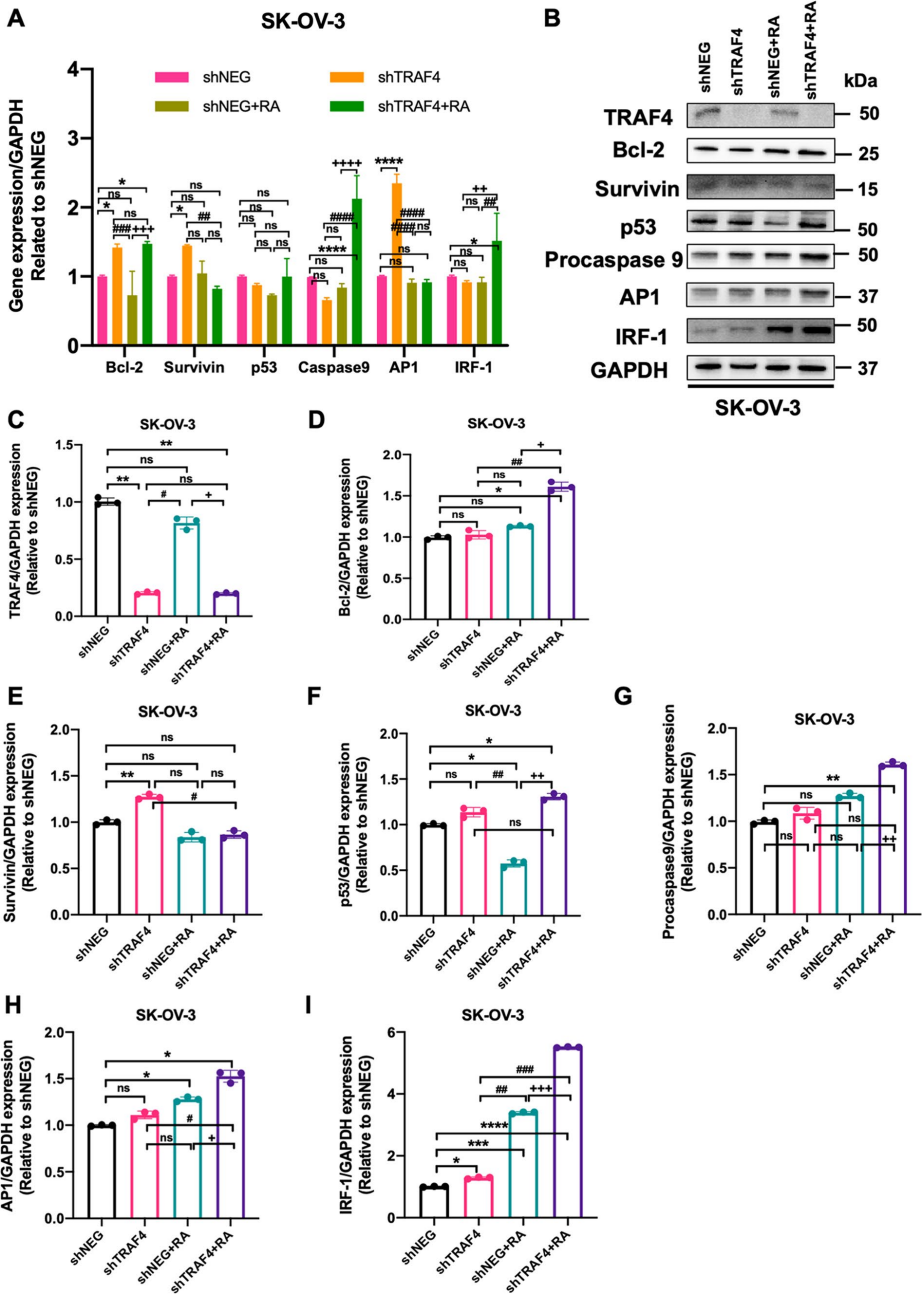
TRAF4 knockdown improved the expression of apoptosis-associated factors in human ovarian cancer SK-OV-3 cells. **A** Relative mRNA levels of apoptosis-associated genes after shTRAF4 knockdown in SK-OV-3 cells with or without retinoic acid (two-way analysis of variance (ANOVA) with Bonferroni’s multiple-comparison test). **B** Protein levels of apoptosis-associated factors after TRAF knockdown (shTRAF4) in SK-OV-3 cells with or without retinoic acid. Relative protein levels of TRAF4 (**C**), Bcl-2 (**D**), Survivin (**E**), p53 (**F**), procaspase 9 (**G**), AP1 (**H**), and IRF-1 (**I**) in SK-OV-3 cells (one-way analysis of variance (ANOVA) with Bonferroni’s multiple-comparison test). All values represent the means of at least three independent experiments ± SD. ns not significant, P < 0.05 was considered statistically significant. (*P < 0.05; **P < 0.01; ***P < 0.001; ****P < 0.0001. ^#^P < 0.05; ^##^P < 0.01; ^###^P < 0.001; ^####^P < 0.0001. ^+^P < 0.05; ^++^P < 0.01; ^+++^P < 0.001; ^++++^P < 0.0001. *P indicates comparisons with shNEG group; ^#^P indicates comparisons with shTRAF4 group; ^+^P indicates comparisons with shNEG + RA group)

As shown in Fig. 5A, TRAF4 inhibition promoted the mRNA levels of Survivin and AP1 in SK-OV-3 cells. Importantly, when retinoic acid treatment was combined with TRAF4 inhibition, the mRNA levels of Caspase 9 and IRF-1 in SK-OV-3 cells were significantly increased compared to the shNEG plus RA group. Additionally, TRAF4 inhibition alone showed no effects on the protein levels of these proteins, except for a slight elevation of Survivin and IRF-1 in SK-OV-3 cells. In contrast, the combination of TRAF4 inhibition with retinoic acid treatment significantly improved the protein levels of both procaspase 9 and IRF-1 compared to the control shNEG group or the shNEG plus RA group. The elevated procaspase 9 and IRF-1 may be triggering apoptosis in human ovarian cancer cell lines (Fig. 5B, I).

**Fig. 6:**
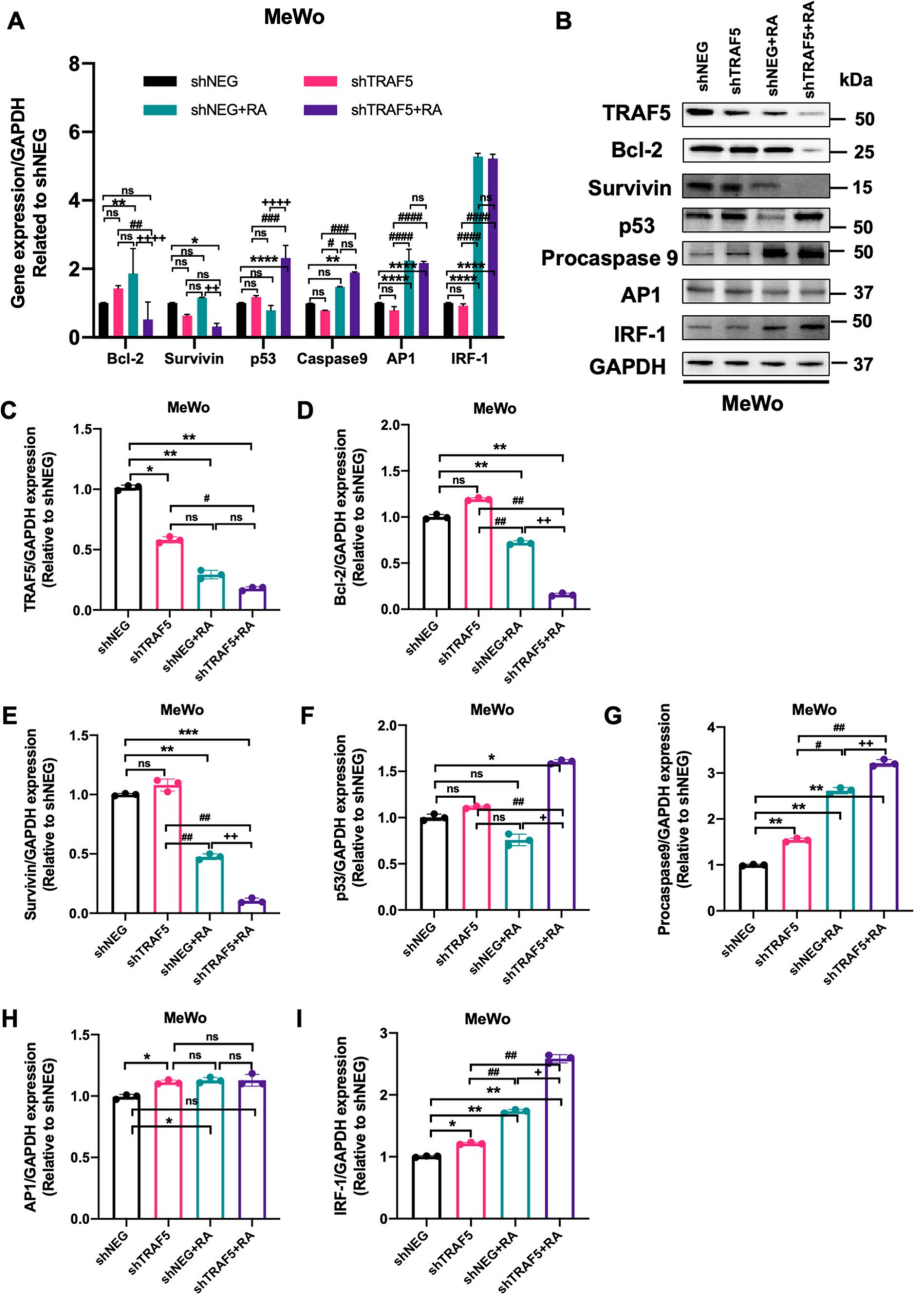
TRAF5 knockdown improved the expression of apoptosis-associated factors in human melanoma MeWo cells. **A** Relative mRNA levels of apoptosis-associated genes after shTRAF5 knockdown in MeWo cells with or without retinoic acid (two-way analysis of variance (ANOVA) with Bonferroni’s multiple-comparison test). **B** Protein levels of apoptosis-associated factors after shTRAF5 knockdown in MeWo cells with or without retinoic acid. Relative protein levels of TRAF5 (**C**), Bcl-2 (**D**), Survivin (**E**), p53 (**F**), procaspase 9 (**G**), AP1(**H**), and IRF-1 (**I**) in MeWo cells (one-way analysis of variance (ANOVA) with Bonferroni’s multiple-comparison test). All values represent the means of at least three independent experiments ± SD. ns not significant, P < 0.05 was considered statistically significant. (*P < 0.05; **P < 0.01; ***P < 0.001; ****P < 0.0001. ^#^P < 0.05; ^##^P < 0.01; ^###^P < 0.001; ^####^P < 0.0001. ^+^P < 0.05; ^++^P < 0.01; ^++++^P < 0.0001. *P indicates comparisons with shNEG group; ^#^P indicates comparisons with shTRAF5 group; ^+^P indicates comparisons with shNEG + RA group)

**Fig. 7:**
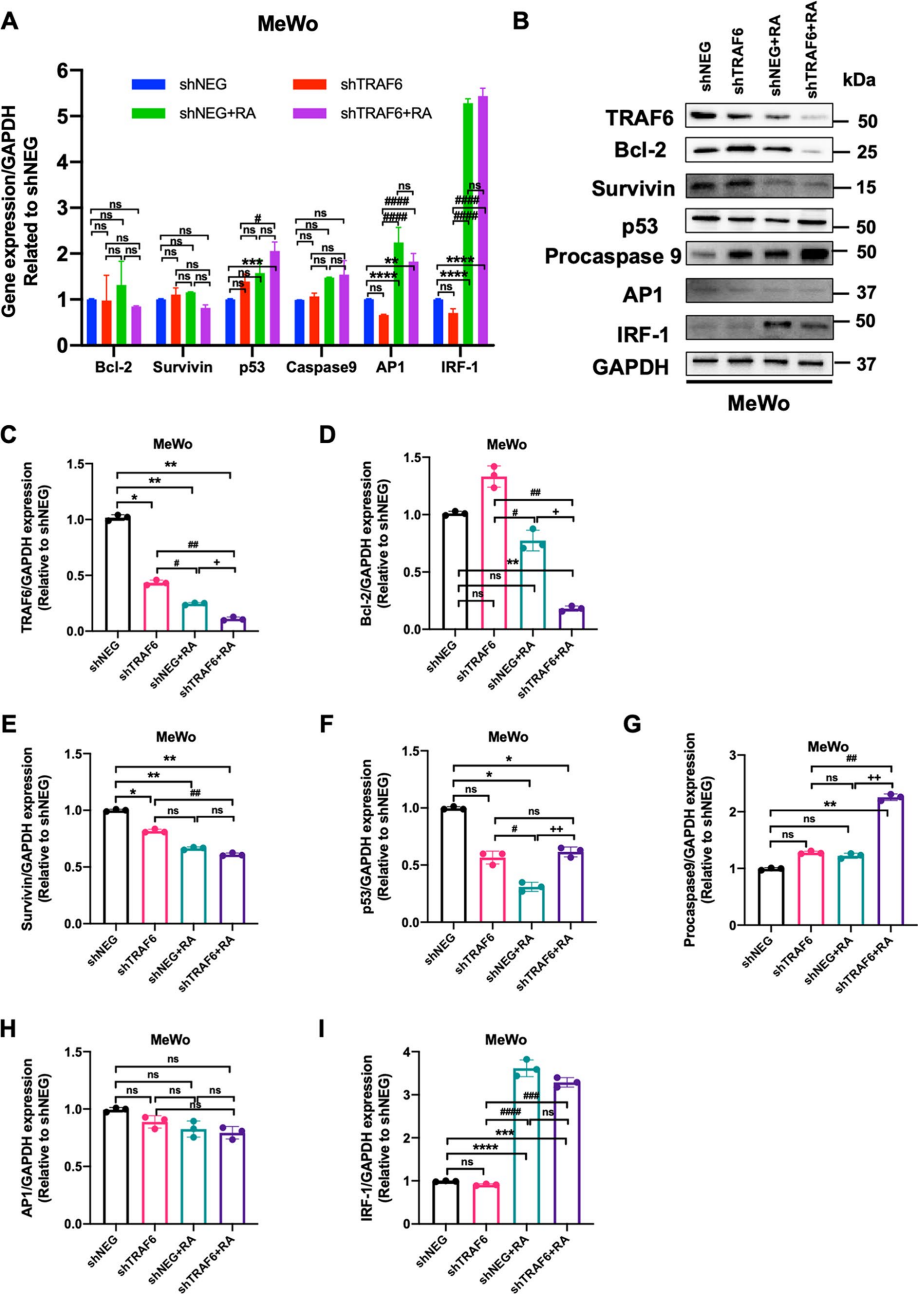
TRAF6 knockdown improved the expression of apoptosis-associated factors in human melanoma MeWo cells. **A** Relative mRNA levels of apoptosis-associated genes after shTRAF6 knockdown in MeWo cells with or without retinoic acid (two-way analysis of variance (ANOVA) with Bonferroni’s multiple-comparison test). **B** Protein levels of apoptosis-associated factors after shTRAF6 knockdown in MeWo cells with or without retinoic acid. Relative protein levels of TRAF6 (**C**), Bcl-2 (**D**), Survivin (**E**), p53 (**F**), procaspase 9 (**G**), AP1 (**H**), and IRF-1 (**I**) in MeWo cells (one-way analysis of variance (ANOVA) with Bonferroni’s multiple-comparison test). All values represent the means of at least three independent experiments ± SD. ns not significant, P < 0.05 was considered statistically significant. (*P < 0.05; **P < 0.01; ***P < 0.001; ****P < 0.0001. ^#^P < 0.05; ^##^P < 0.01; ^###^P < 0.001; ^####^P < 0.0001. ^+^P < 0.05; ^++^P < 0.01. *P indicates comparisons with shNEG group; ^#^P indicates comparisons with shTRAF6 group; ^+^P indicates comparisons with shNEG + RA group)

In MeWo cells, TRAF5 inhibition showed no impact on the mRNA levels of the above proteins. However, compared with the shNEG plus RA group, TRAF4 suppression combined with retinoic acid treatment could dramatically reduce the mRNA levels of Bcl-2 and Survivin, while the mRNA expression of p53 was significantly elevated in MeWo cells (Fig. 6A). Meanwhile, TRAF5 inhibition modestly upregulated the protein levels of procaspase 9, AP1, and IRF-1, while showing no influence on the other examined molecules. Surprisingly, in TRAF5 knockdown MeWo cells with retinoic acid treatment, the protein levels of Bcl-2 and Survivin were drastically diminished, whereas p53, procaspase 9, and IRF-1 were dramatically increased compared with the shNEG + RA group (Fig. 6B, I).

In MeWo cells, neither silencing TRAF6 alone nor TRAF6 inhibition combined with retinoic acid was shown to influence the mRNA expression of the examined molecules associated with apoptosis (Fig. 7A). In contrast, for protein levels, retinoic acid treatment in combination with TRAF6 inhibition significantly decreased the protein levels of Bcl-2, while the protein levels of p53 and procaspase 9 were elevated in the TRAF6 knockdown MeWo cells under retinoic acid treatment, compared to the shNEG plus RA group (Fig. 7B, I).

Taken together, our findings implied that combining retinoic acid with TRAF inhibition enhanced retinoic acid-induced cell apoptosis by improving the expression of several proapoptotic factors while suppressing the expression of the other antiapoptotic factors.

### TRAF inhibition improved retinoic acid sensitivity in human SK-OV-3 and MeWo xenograft tumor models

**Fig. 8:**
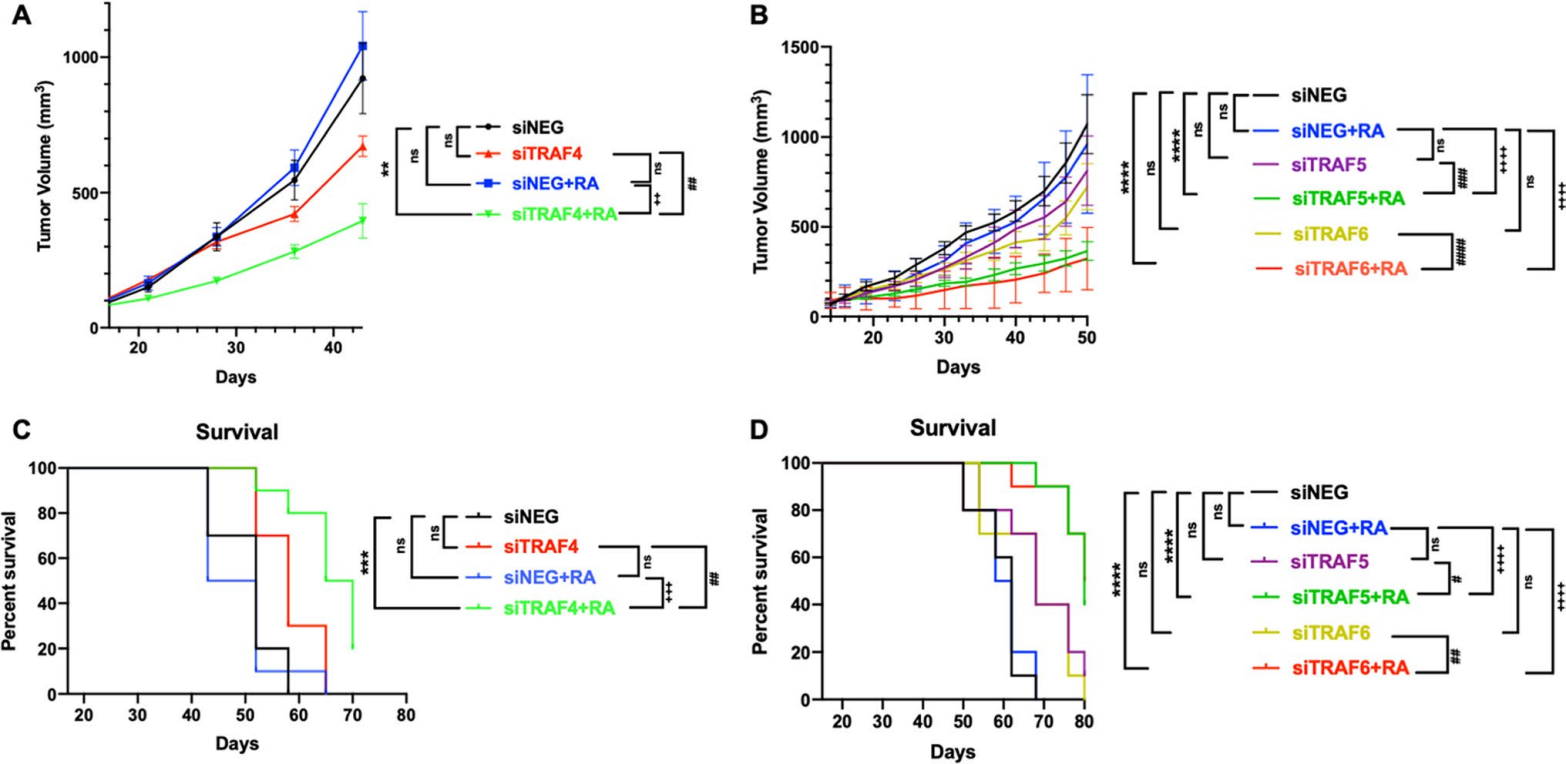
TRAF inhibition improved retinoic acid sensitivity in human SK-OV-3 and MeWo xenograft tumor models. **A** Volumes of SK-OV-3 xenograft tumors from four different treatment groups (n = 10): siNegtive control (siNEG), siTRAF4, siNEG + retinoic acid (RA), and siTRAF4 + retinoic acid (RA). **B** Volumes of MeWo xenograft tumors from six different treatment groups (n = 10): siNegtive control (siNEG), siNEG + retinoic acid (RA), siTRAF5 or siTRAF6, and siTRAF5 or siTRAF6 + retinoic acid (RA). **C** Animal survival curves for the four SK-OV-3 groups. **D** Animal survival curves for the six MeWo groups. Two-way ANOVA with Tukey’s multiple comparisons test was used to calculate the differences in tumor growth curves between treatment groups. Error bars represent the standard error of the mean. Log-rank (Mantel-Cox) test was used to calculate the differences in animal survival between treatment groups. ns not significant, P < 0.05 was considered statistically significant. (^**^P < 0.01; ^***^P < 0.001; ^****^P < 0.0001. ^#^P < 0.05; ^##^P < 0.01. ^###^P < 0.001; ^####^P < 0.0001. ^++^P < 0.01; ^++++^P < 0.0001. ^*^P indicates comparisons with siNEG group; ^#^P indicates comparisons with siTRAF group; ^+^P indicates comparisons with siNEG + RA group)

To further test the combinatorial effects in vivo, the anti-tumor efficacy of TRAF4/5/6 targeted siRNAs, and retinoic acid was evaluated using the human SK-OV-3 and MeWo xenograft models established on nude mice. The four groups of SK-OV-3 mice were administered with siNegtive (intratumoral), siTRAF4 alone (intratumoral), siNegtive (intratumoral) + retinoic acid (gavage), and siTRAF4 (intratumoral) + retinoic acid (gavage) every other day for a total three times per mouse, respectively. The six groups of MeWo mice were administered with siNegtive (intratumoral), siTRAF5 or siTRAF6 alone (intratumoral), siNegtive (intratumoral) + retinoic acid (gavage), and siTRAF5 or siTRAF6 (intratumoral) + retinoic acid (gavage) every other day for a total three times per mouse, respectively. Tumor volume curves showed that the combination of siTRAF + retinoic acid significantly inhibited tumor growth in both two models (Fig. 8A, B and Additional file 2: Tables S4,  Additional file 3: Table S5). A comparison of the animal survival curves also concluded the combination significantly improved the survival compared to the monotherapy groups (Fig. 8C, D). This animal study further confirmed the combinatorial effects of TRAF4/5/6 inhibition plus retinoic acid treatment.

## Discussion

In this study, the tumor necrosis factor receptor-associated factor (TRAF) family members were found to be differentially expressed in various TCGA cancer cohorts and human cancer cell lines. Our results suggested that suppressing TRAF4, TRAF5, or TRAF6 can decrease the proliferation of ovarian cancer and melanoma cells and improve the sensitivity of these cells to retinoic acid treatment, probably by regulating cell apoptosis-related signaling pathways. Based on the findings of this study and our previous studies [36, 37], TRAFs can execute a pivotal role in the development and progression of many cancers and may be an important target for cancer treatment.

The TRAF family has been demonstrated to modulate cell proliferation, apoptosis, and other biological processes. Most TRAFs comprise an N-terminal coiled-coil region (TRAF-N) and a C-terminal β-sandwich (TRAF-C), mediating protein-protein interactions with upstream regulators and downstream effectors [11, 38]. In the current study, even though TRAF7 displayed high mRNA levels and protein levels in the TCGA cancer cohorts and human cancer cell lines, TRAF7 had little effect on the sensitivity of cancer cell lines to retinoic acid, probably due to the lack of a TRAF-C domain. Based on the structures of TRAFs, these proteins function as cytoplasmic junctions that promote transcellular signaling by simultaneously binding to receptors and recruiting adaptor proteins to the cell receptors, including the Toll-like receptors (TLRs), NOD-like receptors (NLRs) and retinoic acid-inducible gene I (RIG-I)-like receptors (RLRs). TRAFs are highly versatile regulators that control a wide range of cellular processes, either acting alone or in combination. In spite of the similarities in the signaling pathways triggered by different TRAF proteins, each TRAF appears to fulfill obligatory and non-redundant physiological roles. Previous studies have confirmed the crucial roles of TRAF3, TRAF2, TRAF5, and TRAF1 in B cell malignancies. Additionally, single nucleotide polymorphisms in TRAF5 were found associated with autoimmune diseases. Abnormal overexpression and gene amplification of TRAF4 and TRAF6 in human carcinomas have been observed to disrupt cell migration, potentially contributing to cancer development [1]. The TRAF family members TRAF4, TRAF5, and TRAF6 are cytoplasmic junction molecules that activate the NF-kappa-B and JNK signaling pathways while regulating cell survival and apoptosis [39, 40]. Targeting TRAFs for drug development has been pursued due to their correlation with various human diseases resulting from their overactivation.

Retinoic acid is a multifunctional molecule that plays a crucial role in cell development, exhibits anti-inflammatory properties, and possesses high anti-cancer potential due to its ability to bind to receptors and regulate gene expression. Previous research has reported that TRAF3 protein could facilitate the retinoic acid-inducible gene (RIG)-I-like receptors (RLRs)-dependent expression of type I IFN by interacting with mitochondrial antiviral signaling (MAVS) [41], while TRAF6 may be recruited by cytosolic retinoic acid-inducible gene I-like receptors to initiate an antiviral response [42, 43]. Although some TRAFs have been shown to interact with retinoic acid-related molecules, their potential role in cancer therapy remains unclear.

In this study, we found that TRAF4, TRAF5, and TRAF6 were upregulated in several TCGA cancer cohorts and cancer cell lines, especially in ovarian cancer and melanoma cell lines, indicating their potential correlation with retinoic acid sensitivity in these cells. We also demonstrated that inhibiting TRAF4, TRAF5, or TRAF6 could suppress cancer cell proliferation, highlighting the critical oncogenic role of TRAFs in carcinogenesis. Targeting TRAF4, TRAF5, or TRAF6 could be a promising strategy for inhibiting ovarian cancer and melanoma cell proliferation.

According to the TCGA melanoma cohort, TRAF6 exhibited a significant upregulation at the mRNA level in melanoma tissues compared to normal tissues. Moreover, TRAF6 was observed to be overexpressed in primary and metastatic melanoma tumors, as well as in some melanoma cell lines [44]. The overexpression and activation of TRAF6 signaling have been shown to enhance melanoma invasion and metastasis [45]. The reason why TRAF6 inhibition only affects MeWo cells in this study could be attributed to the upregulation and hyper-activation of TRAF6 in melanoma. While TRAF6 exhibits similar levels in other cancer cell lines, the entire TRAF6 signaling pathway may not be in a state of hyper-activation.

Moreover, we evaluated the effects of TRAF knockdown on the expression levels of apoptosis-associated proteins in different cancer cell lines and found that under retinoic acid treatment, the proapoptotic protein procaspase 9 significantly increased in TRAF knockdown cells, indicating a synergistic effect of TRAF knockdown with retinoic acid in triggering cell apoptosis. Previous research reported that retinoic acid activates the intrinsic activation of p53 by a novel mechanism independent of effects on p53 stability [46]. In this study, we noticed a decrease in p53 levels in the SK-OV-3 and MeWo cell lines following RA treatment, and the mechanism behind this effect is currently unknown. It is possible that the impact of retinoic acid treatment on p53 expression levels varies between cell lines, as a previous study reported that RA treatment reduced p53 expression levels in the SK-N-FI neuroblastoma cells but not in another neuroblastoma cell line, SK-N-Be (2) [47]. In addition, TRAF4, TRAF5, and TRAF6 possess E3 ubiquitin ligase activities that are involved in the degradation of many signaling molecules through ubiquitination events. Therefore, it would be important to define if inhibiting their E3 ligase activity is sufficient to potentiate RA treatment.

## Conclusions

In conclusion, our study demonstrates that reducing the expression of TRAF4, TRAF5, or TRAF6 significantly improves the sensitivity of human ovarian cancer or melanoma cells to retinoic acid. Combining TRAF inhibition with retinoic acid treatment enhances cell apoptosis, leading to suppressing the progression of ovarian cancer or melanoma. These findings indicate that combining TRAF inhibition with retinoic acid treatment could potentially be an effective therapy for human cancer.

## Supplementary Information

**Additional file 1:** **Table S1. **Antibodies,siRNA and lentiviral particles. Table S2. The target sequences used for siRNA orshRNA knockdown. Table S3. Thesequences of Q-PCR primers. **Figure S1. **Effects of siRNA mediated TRAFsknockdown on retinoic acid sensitivity in human ovarian cancer SK-OV-3 cells.TRAF1.TRAF2.TRAF3.TRAF5.TRAF6.TRAF7. All values represent the means of at least threeindependent experiments ± standard deviation.  **FigureS2. **Effects of siRNA mediated TRAFs knockdown on retinoic acid sensitivity in humanmelanoma MeWo cells.TRAF1.TRAF2.TRAF3.TRAF4.TRAF7. All values represent the means of at least threeindependent experiments ± standard deviation. **FigureS3. **Effects of siRNA mediated TRAFs knockdown on retinoic acid sensitivity in humanglioblastoma U251 cells.TRAF1.TRAF2.TRAF3.TRAF4.TRAF5.TRAF6.TRAF7. Allvalues represent the means of at least three independent experiments ± standarddeviation. **FigureS4. **Effects of siRNA mediated TRAFs knockdown on retinoic acid sensitivity in humanprostate cancer 22RV1 cells.TRAF1.TRAF2.TRAF3.TRAF4.TRAF5.TRAF6.TRAF7. Allvalues represent the means of at least three independent experiments ± standarddeviation.**FigureS5.** Effects of siRNA mediated TRAFs knockdown on retinoic acid sensitivity in humanbreast cancer BT549 cells.TRAF1.TRAF2.TRAF3.TRAF4.TRAF5.TRAF6.TRAF7. Allvalues represent the means of at least three independent experiments ± standarddeviation.  **FigureS6. **Original pictures of TRAF1/TRAF2/TRAF3/TRAF5/TRAF6/TRAF7protein blotted in various cancer cell lines. **Figure S7.** Originalpictures of TRAF4/ TRAF5/TRAF6 knockdown. **FigureS8.** Original pictures of TRAF4 knockdown andapoptosis-related proteins. **Figure S9. **Originalpictures of TRAF5 knockdown and apoptosis-related proteins. **Figure S10. **Originalpictures of TRAF6 knockdown and apoptosis-related proteins. 

**Additional file 2: Table S4.** SK-OV-3  tumor volumes (mm3)

**Additional file 3: Table S5.** MeWo tumor volumes (mm3)

## Data Availability

All data generated and analyzed during this study are available from the corresponding author upon reasonable request.
