# Supplementary material for: TRAF inhibition drives cancer cell apoptosis and improves retinoic acid sensitivity in multiple cancers models
Source: Discov Oncol. 2023 Jun 30;14:117. doi: 10.1007/s12672-023-00703-5 (PMC10313640; doi:10.1007/s12672-023-00703-5)
Supplement: Supplementary file 1 — Additional file 1: Table S1. Antibodies,siRNA and lentiviral particles. Table S2. The target sequences used for siRNA orshRNA knockdown. Table S3. Thesequences of Q-PCR primers. Figure S1. Effects of siRNA mediated TRAFsknockdown on retinoic acid sensitivity in human ovarian cancer SK-OV-3 cells.TRAF1.TRAF2.TRAF3.TRAF5.TRAF6.TRAF7. All values represent the means of at least threeindependent experiments ± standard deviation. FigureS2. Effects of siRNA mediated TRAFs knockdown on retinoic acid sensitivity in humanmelanoma MeWo cells.TRAF1.TRAF2.TRAF3.TRAF4.TRAF7. All values represent the means of at least threeindependent experiments ± standard deviation. FigureS3. Effects of siRNA mediated TRAFs knockdown on retinoic acid sensitivity in humanglioblastoma U251 cells.TRAF1.TRAF2.TRAF3.TRAF4.TRAF5.TRAF6.TRAF7. Allvalues represent the means of at least three independent experiments ± standarddeviation. FigureS4. Effects of siRNA mediated TRAFs knockdown on retinoic acid sensitivity in humanprostate cancer 22RV1 cells.TRAF1.TRAF2.TRAF3.TRAF4.TRAF5.TRAF6.TRAF7. Allvalues represent the means of at least three independent experiments ± standarddeviation.FigureS5. Effects of siRNA mediated TRAFs knockdown on retinoic acid sensitivity in humanbreast cancer BT549 cells.TRAF1.TRAF2.TRAF3.TRAF4.TRAF5.TRAF6.TRAF7. Allvalues represent the means of at least three independent experiments ± standarddeviation. FigureS6. Original pictures of TRAF1/TRAF2/TRAF3/TRAF5/TRAF6/TRAF7protein blotted in various cancer cell lines. Figure S7. Originalpictures of TRAF4/ TRAF5/TRAF6 knockdown. FigureS8. Original pictures of TRAF4 knockdown andapoptosis-related proteins. Figure S9. Originalpictures of TRAF5 knockdown and apoptosis-related proteins. Figure S10. Originalpictures of TRAF6 knockdown and apoptosis-related proteins. [file 12672_2023_703_MOESM1_ESM.docx]

**Table S1. Antibodies, siRNA and lentiviral particles**

| Products | Company | Catalog number |
| --- | --- | --- |
| Retinoic acid | Sigma, St. Louis, MO, USA | R2625 |
| TRAF1 antibody | Cell signaling Technology, Danvers, MA, USA | 4715 |
| TRAF2 antibody | Cell signaling Technology, Danvers, MA, USA | 4724 |
| TRAF3 antibody | Cell signaling Technology, Danvers, MA, USA | 4729 |
| TRAF4 antibody | PROTEINTECH NORTH AMERICA, Rosemont, IL, USA | 66755-1-Ig |
| TRAF5 antibody | Cell signaling Technology, Danvers, MA, USA | 41658 |
| TRAF6 antibody | Cell signaling Technology, Danvers, MA, USA | 8028 |
| TRAF7 antibody | PROTEINTECH NORTH AMERICA, Rosemont, IL, USA | 11780-1-AP |
| BCL2 antibody | Cell signaling Technology, Danvers, MA, USA | 15071 |
| Survivin antibody | PROTEINTECH NORTH AMERICA, Rosemont, IL, USA | 10508-1-AP |
| p53 antibody | PROTEINTECH NORTH AMERICA, Rosemont, IL, USA | 10442-1-AP |
| Caspase9 antibody | PROTEINTECH NORTH AMERICA, Rosemont, IL, USA | 66169-1-Ig |
| Activator protein 1  (AP-1) antibody | PROTEINTECH NORTH AMERICA, Rosemont, IL, USA | 22114-1-AP |
| Interferon regulatory factor-1  (IRF1) antibody | Cell signaling Technology, Danvers, MA, USA | 8478 |
| GAPDH antibody | Cell signaling Technology, Danvers, MA, USA | 2118 |
| Anti-rabbit IgG, HRP-linked Antibody | Cell signaling Technology, Danvers, MA, USA | 7074 |
| Anti-mouse IgG, HRP-linked Antibody | Cell signaling Technology, Danvers, MA, USA | 7076 |
| Control shRNA Lentiviral Particles-A | Santa Cruz Biotechnology, Santa Cruz, CA, USA | sc-108080 |
| TRAF4 shRNA (h) Lentiviral Particles | Santa Cruz Biotechnology, Santa Cruz, CA, USA | sc-36713-V |
| TRAF5 shRNA (h) Lentiviral Particles | Santa Cruz Biotechnology, Santa Cruz, CA, USA | sc-36715-V |
| TRAF6 shRNA (h) Lentiviral Particles | Santa Cruz Biotechnology, Santa Cruz, CA, USA | sc-36717-V |
| siGENOME non-targeting siRNA Control Pools | [Horizon](https://horizondiscovery.com/en/gene-modulation/knockdown/sirna/products/sigenome-sirna-reagents) Discovery, [Waterbeach, United Kingdom](https://www.google.com/search?q=Waterbeach&stick=H4sIAAAAAAAAAOPgE-LSz9U3qEoqzjHOUeIEsc2Mi-OTtLSyk63084vSE_MyqxJLMvPzUDhWGamJKYWliUUlqUXFi1i5whOBjKTUxOSMHayMAOSD6DtVAAAA&sa=X&ved=2ahUKEwipn8yc0ZP0AhXQkWoFHaYMA5oQmxMoAXoECEAQAw) | D-001206-13-5 |
| siGENMONE SMARTpool TRAF1 siRNA | [Horizon](https://horizondiscovery.com/en/gene-modulation/knockdown/sirna/products/sigenome-sirna-reagents) Discovery, [Waterbeach, United Kingdom](https://www.google.com/search?q=Waterbeach&stick=H4sIAAAAAAAAAOPgE-LSz9U3qEoqzjHOUeIEsc2Mi-OTtLSyk63084vSE_MyqxJLMvPzUDhWGamJKYWliUUlqUXFi1i5whOBjKTUxOSMHayMAOSD6DtVAAAA&sa=X&ved=2ahUKEwipn8yc0ZP0AhXQkWoFHaYMA5oQmxMoAXoECEAQAw) | M-017438-01-0005 |
| siGENMONE SMARTpool TRAF2 siRNA | [Horizon](https://horizondiscovery.com/en/gene-modulation/knockdown/sirna/products/sigenome-sirna-reagents) Discovery, [Waterbeach, United Kingdom](https://www.google.com/search?q=Waterbeach&stick=H4sIAAAAAAAAAOPgE-LSz9U3qEoqzjHOUeIEsc2Mi-OTtLSyk63084vSE_MyqxJLMvPzUDhWGamJKYWliUUlqUXFi1i5whOBjKTUxOSMHayMAOSD6DtVAAAA&sa=X&ved=2ahUKEwipn8yc0ZP0AhXQkWoFHaYMA5oQmxMoAXoECEAQAw) | M-005198-00-0005 |
| siGENMONE SMARTpool TRAF3 siRNA | [Horizon](https://horizondiscovery.com/en/gene-modulation/knockdown/sirna/products/sigenome-sirna-reagents) Discovery, [Waterbeach, United Kingdom](https://www.google.com/search?q=Waterbeach&stick=H4sIAAAAAAAAAOPgE-LSz9U3qEoqzjHOUeIEsc2Mi-OTtLSyk63084vSE_MyqxJLMvPzUDhWGamJKYWliUUlqUXFi1i5whOBjKTUxOSMHayMAOSD6DtVAAAA&sa=X&ved=2ahUKEwipn8yc0ZP0AhXQkWoFHaYMA5oQmxMoAXoECEAQAw) | M-005252-02-0005 |
| siGENMONE SMARTpool TRAF4 siRNA | [Horizon](https://horizondiscovery.com/en/gene-modulation/knockdown/sirna/products/sigenome-sirna-reagents) Discovery, [Waterbeach, United Kingdom](https://www.google.com/search?q=Waterbeach&stick=H4sIAAAAAAAAAOPgE-LSz9U3qEoqzjHOUeIEsc2Mi-OTtLSyk63084vSE_MyqxJLMvPzUDhWGamJKYWliUUlqUXFi1i5whOBjKTUxOSMHayMAOSD6DtVAAAA&sa=X&ved=2ahUKEwipn8yc0ZP0AhXQkWoFHaYMA5oQmxMoAXoECEAQAw) | M-006908-01-0005 |
| siGENMONE SMARTpool TRAF5 siRNA | [Horizon](https://horizondiscovery.com/en/gene-modulation/knockdown/sirna/products/sigenome-sirna-reagents) Discovery, [Waterbeach, United Kingdom](https://www.google.com/search?q=Waterbeach&stick=H4sIAAAAAAAAAOPgE-LSz9U3qEoqzjHOUeIEsc2Mi-OTtLSyk63084vSE_MyqxJLMvPzUDhWGamJKYWliUUlqUXFi1i5whOBjKTUxOSMHayMAOSD6DtVAAAA&sa=X&ved=2ahUKEwipn8yc0ZP0AhXQkWoFHaYMA5oQmxMoAXoECEAQAw) | M-006568-01-0005 |
| siGENMONE SMARTpool TRAF6 siRNA | [Horizon](https://horizondiscovery.com/en/gene-modulation/knockdown/sirna/products/sigenome-sirna-reagents) Discovery, [Waterbeach, United Kingdom](https://www.google.com/search?q=Waterbeach&stick=H4sIAAAAAAAAAOPgE-LSz9U3qEoqzjHOUeIEsc2Mi-OTtLSyk63084vSE_MyqxJLMvPzUDhWGamJKYWliUUlqUXFi1i5whOBjKTUxOSMHayMAOSD6DtVAAAA&sa=X&ved=2ahUKEwipn8yc0ZP0AhXQkWoFHaYMA5oQmxMoAXoECEAQAw) | M-004712-00-0005 |
| siGENMONE SMARTpool TRAF7 siRNA | [Horizon](https://horizondiscovery.com/en/gene-modulation/knockdown/sirna/products/sigenome-sirna-reagents) Discovery, [Waterbeach, United Kingdom](https://www.google.com/search?q=Waterbeach&stick=H4sIAAAAAAAAAOPgE-LSz9U3qEoqzjHOUeIEsc2Mi-OTtLSyk63084vSE_MyqxJLMvPzUDhWGamJKYWliUUlqUXFi1i5whOBjKTUxOSMHayMAOSD6DtVAAAA&sa=X&ved=2ahUKEwipn8yc0ZP0AhXQkWoFHaYMA5oQmxMoAXoECEAQAw) | M-007086-00-0005 |

**Table S2. The target sequences used for siRNA or shRNA knockdown**

| Products | Company | Sequences (5′ → 3′) |
| --- | --- | --- |
| siGENMONE SMARTpool TRAF1 siRNA | [Horizon](https://horizondiscovery.com/en/gene-modulation/knockdown/sirna/products/sigenome-sirna-reagents) Discovery, [Waterbeach, United Kingdom](https://www.google.com/search?q=Waterbeach&stick=H4sIAAAAAAAAAOPgE-LSz9U3qEoqzjHOUeIEsc2Mi-OTtLSyk63084vSE_MyqxJLMvPzUDhWGamJKYWliUUlqUXFi1i5whOBjKTUxOSMHayMAOSD6DtVAAAA&sa=X&ved=2ahUKEwipn8yc0ZP0AhXQkWoFHaYMA5oQmxMoAXoECEAQAw) | 1. CCAGAGAGAAGAAGAUAAA |
|  |  | 2. GGAAGUGACUUCUCCUUGA |
|  |  | 3. GGUCACCCAGACACUCCAA |
|  |  | 4. AGAGAAGAAGAUAAAGUCA |
| siGENMONE SMARTpool TRAF2  siRNA | [Horizon](https://horizondiscovery.com/en/gene-modulation/knockdown/sirna/products/sigenome-sirna-reagents) Discovery, [Waterbeach, United Kingdom](https://www.google.com/search?q=Waterbeach&stick=H4sIAAAAAAAAAOPgE-LSz9U3qEoqzjHOUeIEsc2Mi-OTtLSyk63084vSE_MyqxJLMvPzUDhWGamJKYWliUUlqUXFi1i5whOBjKTUxOSMHayMAOSD6DtVAAAA&sa=X&ved=2ahUKEwipn8yc0ZP0AhXQkWoFHaYMA5oQmxMoAXoECEAQAw) | 1. GGACCAAGACAAGAUUGAA |
|  |  | 2. GCGAGAGCCUGGAGAAGAA |
|  |  | 3. GCUGCGGAGCAGACGUGAA |
|  |  | 4. CGAAGACAGAGUUAUUAAA |
| siGENMONE SMARTpool TRAF3 siRNA | [Horizon](https://horizondiscovery.com/en/gene-modulation/knockdown/sirna/products/sigenome-sirna-reagents) Discovery, [Waterbeach, United Kingdom](https://www.google.com/search?q=Waterbeach&stick=H4sIAAAAAAAAAOPgE-LSz9U3qEoqzjHOUeIEsc2Mi-OTtLSyk63084vSE_MyqxJLMvPzUDhWGamJKYWliUUlqUXFi1i5whOBjKTUxOSMHayMAOSD6DtVAAAA&sa=X&ved=2ahUKEwipn8yc0ZP0AhXQkWoFHaYMA5oQmxMoAXoECEAQAw) | 1. GGUUGUGCAGAGCAGUUAA |
|  |  | 2. GGAAGAUUCGCGACUACAA |
|  |  | 3. GGACAAACCAGCAGAUCAA |
|  |  | 4. GUUGCAGAAUGAAAGUGUA |
| siGENMONE SMARTpool TRAF4 siRNA | [Horizon](https://horizondiscovery.com/en/gene-modulation/knockdown/sirna/products/sigenome-sirna-reagents) Discovery, [Waterbeach, United Kingdom](https://www.google.com/search?q=Waterbeach&stick=H4sIAAAAAAAAAOPgE-LSz9U3qEoqzjHOUeIEsc2Mi-OTtLSyk63084vSE_MyqxJLMvPzUDhWGamJKYWliUUlqUXFi1i5whOBjKTUxOSMHayMAOSD6DtVAAAA&sa=X&ved=2ahUKEwipn8yc0ZP0AhXQkWoFHaYMA5oQmxMoAXoECEAQAw) | 1. GAAACUAUGUGCGGGAUGA |
|  |  | 2. UGAUCUACCUGCACACUUG |
|  |  | 3. GGCCACCGUUUCUGCGAUA |
|  |  | 4. CAUCCGUGCUGCUGUUGAA |
| siGENMONE SMARTpool TRAF5 siRNA | [Horizon](https://horizondiscovery.com/en/gene-modulation/knockdown/sirna/products/sigenome-sirna-reagents) Discovery, [Waterbeach, United Kingdom](https://www.google.com/search?q=Waterbeach&stick=H4sIAAAAAAAAAOPgE-LSz9U3qEoqzjHOUeIEsc2Mi-OTtLSyk63084vSE_MyqxJLMvPzUDhWGamJKYWliUUlqUXFi1i5whOBjKTUxOSMHayMAOSD6DtVAAAA&sa=X&ved=2ahUKEwipn8yc0ZP0AhXQkWoFHaYMA5oQmxMoAXoECEAQAw) | 1. GUGCGAAGAUUAUUCUAAA |
|  |  | 2. GCAGCUAGCAGAAACUAUA |
|  |  | 3. GGAAGGUGACAGAUUACAA |
|  |  | 4. CAACCUGUGCAGUGUUCUA |
| siGENMONE SMARTpool TRAF6 siRNA | [Horizon](https://horizondiscovery.com/en/gene-modulation/knockdown/sirna/products/sigenome-sirna-reagents) Discovery, [Waterbeach, United Kingdom](https://www.google.com/search?q=Waterbeach&stick=H4sIAAAAAAAAAOPgE-LSz9U3qEoqzjHOUeIEsc2Mi-OTtLSyk63084vSE_MyqxJLMvPzUDhWGamJKYWliUUlqUXFi1i5whOBjKTUxOSMHayMAOSD6DtVAAAA&sa=X&ved=2ahUKEwipn8yc0ZP0AhXQkWoFHaYMA5oQmxMoAXoECEAQAw) | 1. GCAAGUGAUAAUCAAGUUA |
|  |  | 2. GGUGAAAUGUCCAAAUGAA |
|  |  | 3. CAGAGCAAGUGAUAAUCAA |
|  |  | 4. CAAGUGAUUUGCACAAAUA |
| siGENMONE SMARTpool TRAF7 siRNA | [Horizon](https://horizondiscovery.com/en/gene-modulation/knockdown/sirna/products/sigenome-sirna-reagents) Discovery, [Waterbeach, United Kingdom](https://www.google.com/search?q=Waterbeach&stick=H4sIAAAAAAAAAOPgE-LSz9U3qEoqzjHOUeIEsc2Mi-OTtLSyk63084vSE_MyqxJLMvPzUDhWGamJKYWliUUlqUXFi1i5whOBjKTUxOSMHayMAOSD6DtVAAAA&sa=X&ved=2ahUKEwipn8yc0ZP0AhXQkWoFHaYMA5oQmxMoAXoECEAQAw) | 1. GGAGAAGAUCGACCAGCUA |
|  |  | 2. GGAGUGCGAGCACAUCAAA |
|  |  | 3. GACCGUAGAUGCCUCUCUA |
|  |  | 4. AGGAGUGCGAGCACAUCAA |
| TRAF4 shRNA (h) | Santa Cruz Biotechnology, Santa Cruz, CA, USA | Sense1: UCAGUGAAGGAGUCUUCAA |
|  |  | Antisense1: UUGAAGACUCCUUCACUGA |
|  |  | Sense2: CUGGACUAUGCCAAGAUCU |
|  |  | Antisense 2: AGAUCUUGGCAUAGUCCAG |
|  |  | Sense3: GGCUUUGGUUAUCCCAAGU |
|  |  | Antisense 3: ACUUGGGAUAACCAAAGCC |
| TRAF5 shRNA (h) | Santa Cruz Biotechnology, Santa Cruz, CA, USA | Sense1: GCUGGAGGGUACUUGCUAU |
|  |  | Antisense1:AUAGCAAGUACCCUCCAGC |
|  |  | Sense2: GCAGCUAGCAGAAACUAUA |
|  |  | Antisense 2: UAUAGUUUCUGCUAGCUGC |
|  |  | Sense3: GCAAUGCUCCUGGAUGUAA |
|  |  | Antisense 3: UUACAUCCAGGAGCAUUGC |
| TRAF6 shRNA (h) | Santa Cruz Biotechnology, Santa Cruz, CA, USA | Sense1: GGUACAAUACGCCUUACAA |
|  |  | Antisense1: UUGUAAGGCGUAUUGUACC |
|  |  | Sense2: GCAACUUUGGAAUGCAUUU |
|  |  | Antisense 2: AAAUGCAUUCCAAAGUUGC |
|  |  | Sense3: GCCCAGGCUGUUCAUAGUUU |
|  |  | Antisense 3: AAACUAUGAACAGCCUGGGC |

SMARTpool: A mixture of 4 siRNA provided as a single reagent.

TRAF4 shRNA (h): a pool of 3 different shRNA plasmids.

TRAF5 shRNA (h): a pool of 3 different shRNA plasmids.

TRAF6 shRNA (h): a pool of 3 different shRNA plasmids.

**Table S3. The sequences of Q-PCR primers.**

| BCL2 Primer | F: 5’- ATCGCCCTGTGGATGACTGAGT-3’ |
| --- | --- |
|  | R: 5’- GCCAGGAGAAATCAAACAGAGGC-3’ |
| Survivin Primer | F: 5’- CCACTGAGAACGAGCCAGACTT-3’ |
|  | R: 5’- GTATTACAGGCGTAAGCCACCG-3’ |
| p53 Primer | F: 5’-CCTCAGCATCTTATCCGAGTGG-3’ |
|  | R: 5’-TGGATGGTGGTACAGTCAGAGC-3’ |
| Caspase9 Primer | F: 5’- GTTTGAGGACCTTCGACCAGCT-3’ |
|  | R: 5’- CAACGTACCAGGAGCCACTCTT-3’ |
| Activator protein 1  (AP-1) Primer | F: 5’- CCTTGAAAGCTCAGAACTCGGAG-3’ |
|  | R: 5’- TGCTGCGTTAGCATGAGTTGGC-3’ |
| Interferon regulatory factor-1  (IRF1) Primer | F: 5’- GAGGAGGTGAAAGACCAGAGCA-3’ |
|  | R: 5’- TAGCATCTCGGCTGGACTTCGA-3’ |
| GAPDH Q-PCR Primer | F: 5’-GTCTCCTCTGACTTCAACAGCG-3’ |
|  | R: 5’-ACCACCCTGTTGCTGTAGCCAA-3’ |

**Figures**

**
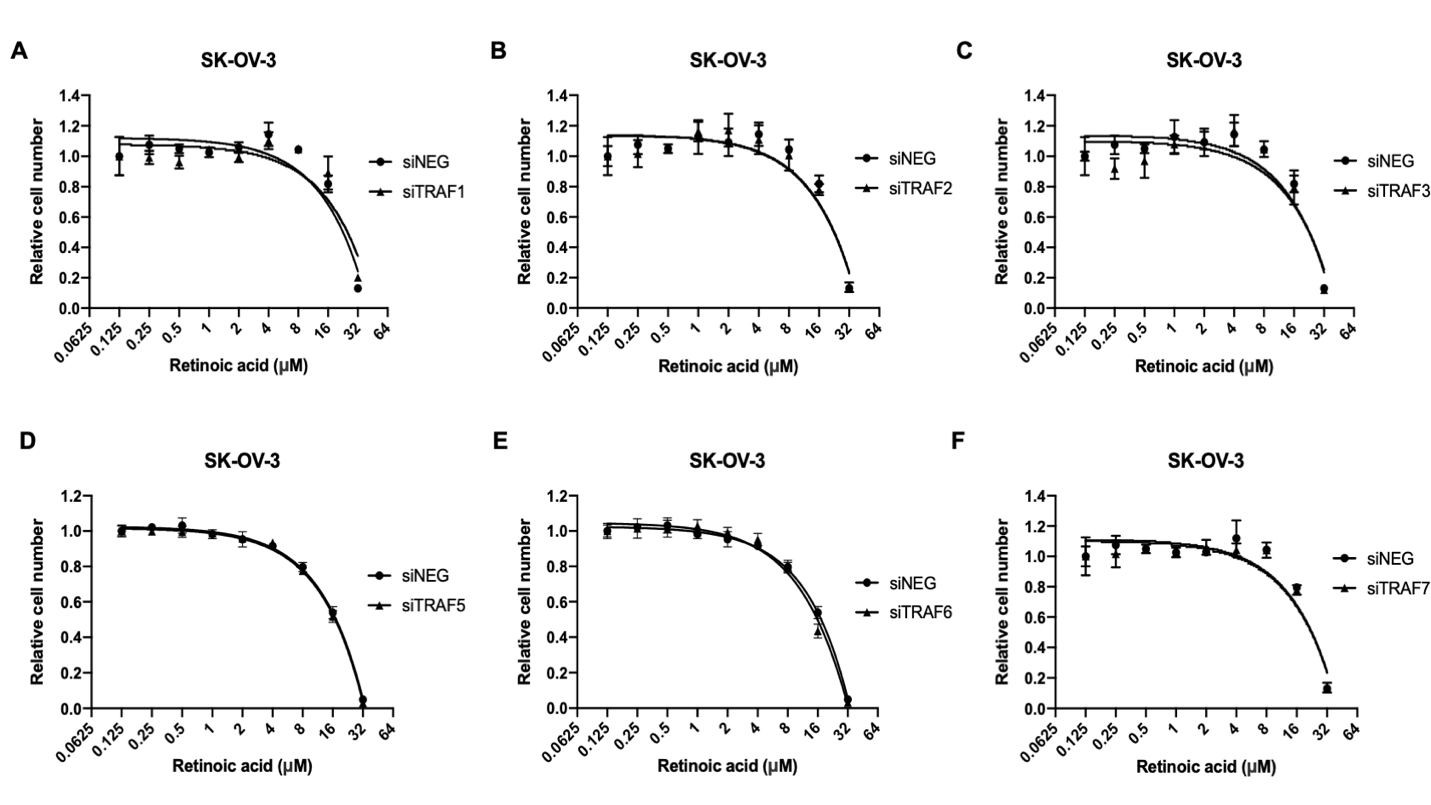
**

**Figure S1. Effects of siRNA mediated** **TRAFs knockdown on retinoic acid sensitivity in human ovarian cancer SK-OV-3 cells**. (A) TRAF1. (B) TRAF2. (C) TRAF3. (D) TRAF5. (E) TRAF6. (F) TRAF7. All values represent the means of at least three independent experiments ± standard deviation (SD).

**
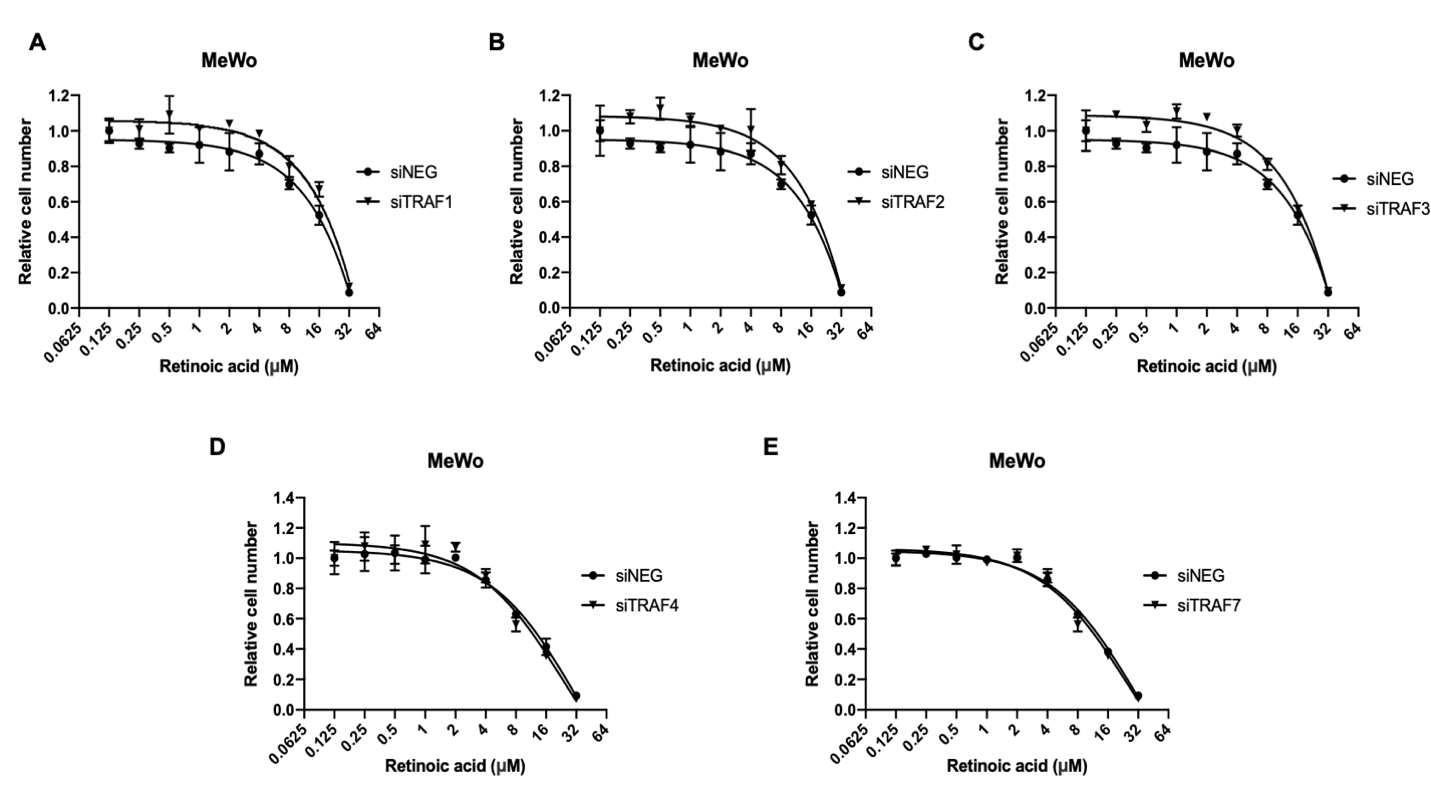
**

**Figure S2. Effects of siRNA mediated** **TRAFs knockdown on retinoic acid sensitivity in human melanoma MeWo cells.** (A) TRAF1. (B) TRAF2. (C) TRAF3. (D) TRAF4. (E) TRAF7. All values represent the means of at least three independent experiments ± standard deviation (SD).

**
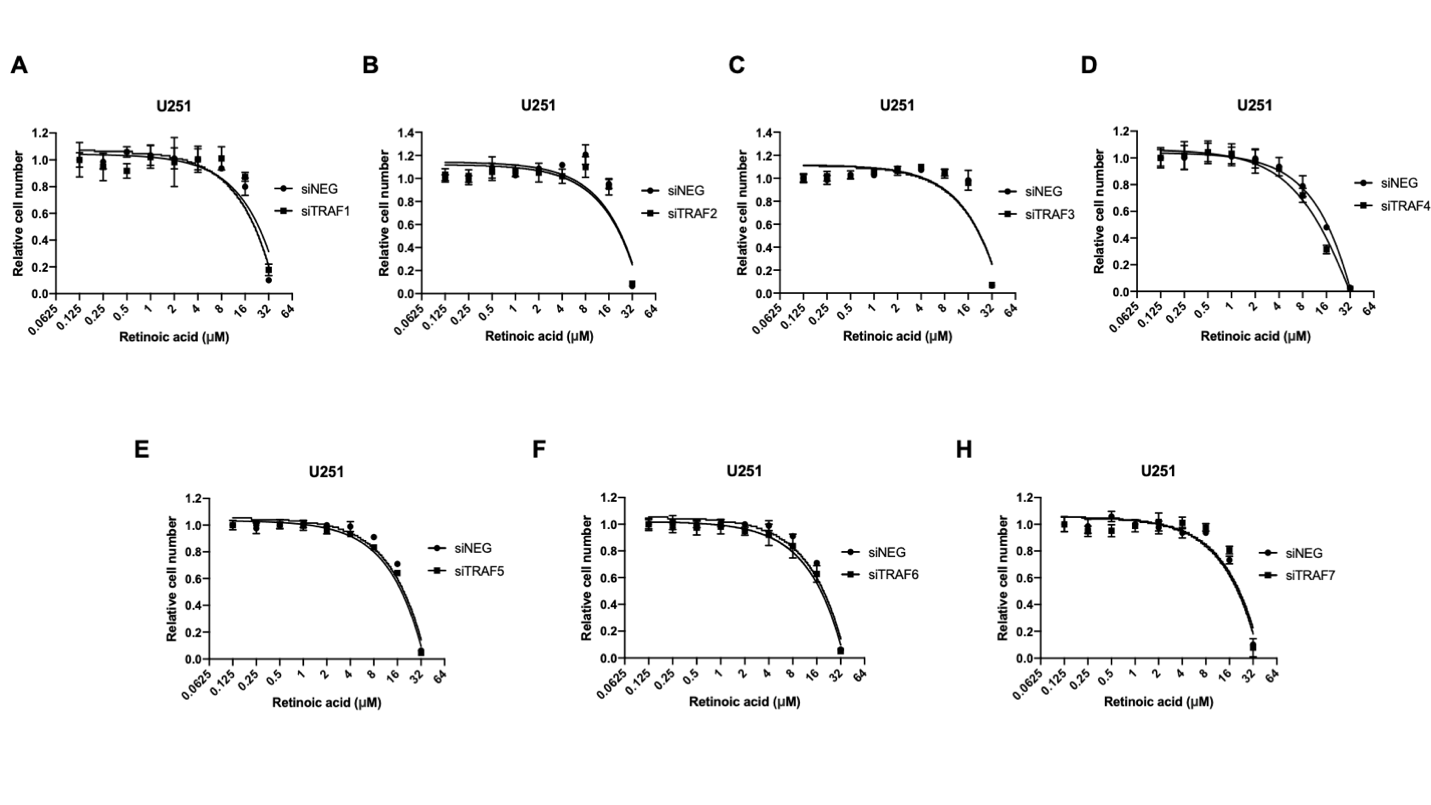
**

**Figure S3. Effects of siRNA mediated** **TRAFs knockdown on retinoic acid sensitivity in human glioblastoma U251 cells.** (A) TRAF1. (B) TRAF2. (C) TRAF3. (D) TRAF4. (E) TRAF5. (F) TRAF6. (H) TRAF7. All values represent the means of at least three independent experiments ± standard deviation (SD).

**
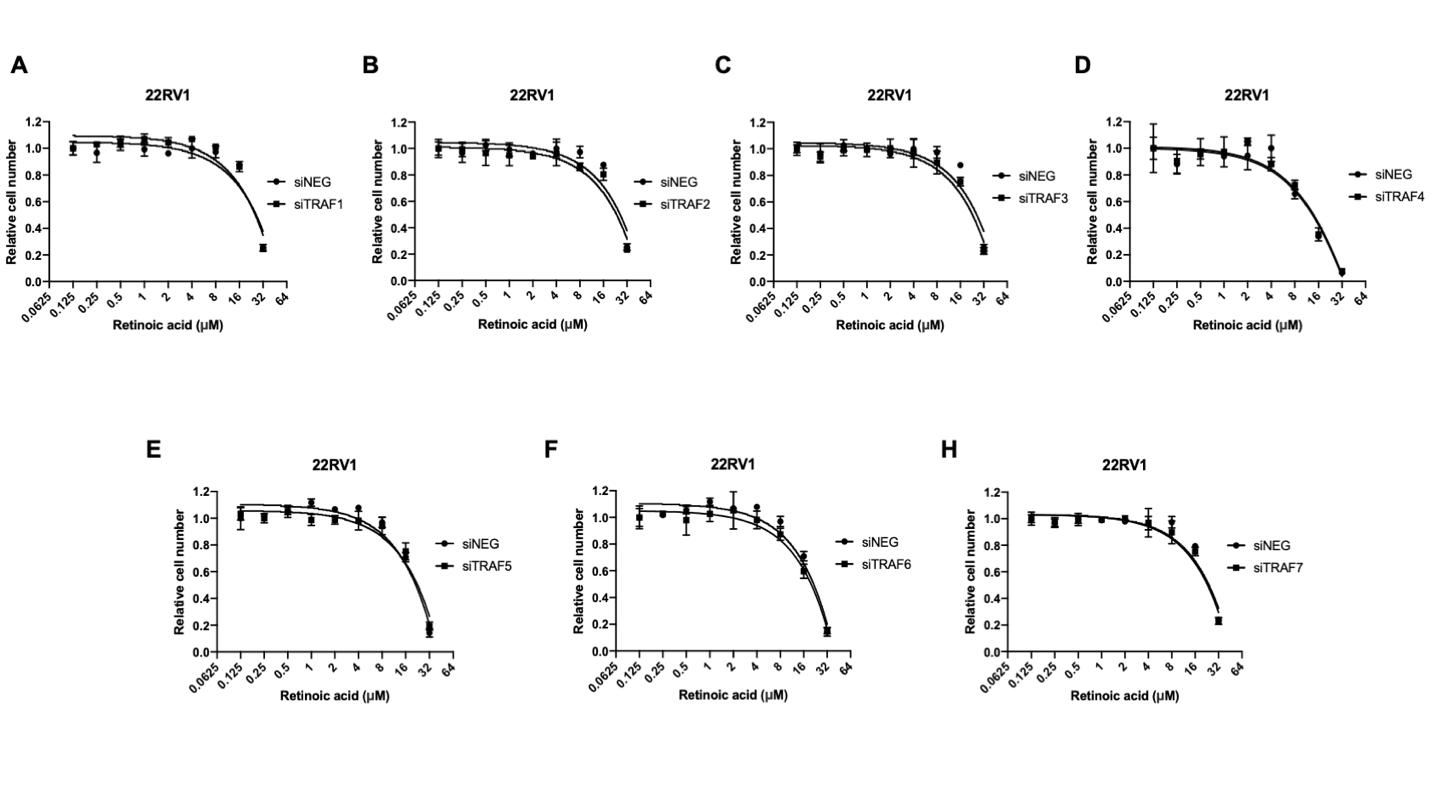
**

**Figure S4. Effects of siRNA mediated** **TRAFs knockdown on retinoic acid sensitivity in human prostate cancer 22RV1 cells.** (A) TRAF1. (B) TRAF2. (C) TRAF3. (D) TRAF4. (E) TRAF5. (F) TRAF6. (H) TRAF7. All values represent the means of at least three independent experiments ± standard deviation (SD).

**
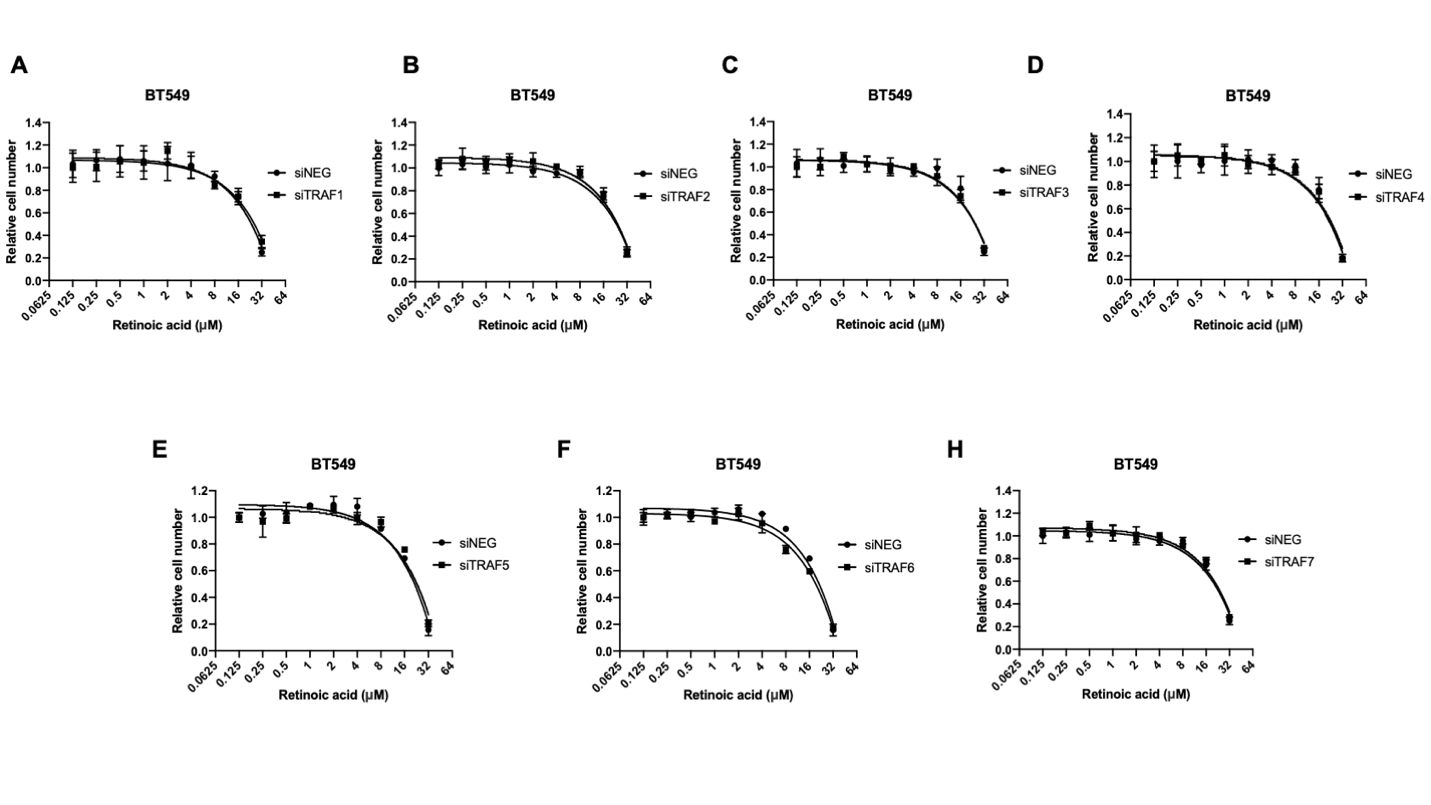
**

**Figure S5. Effects of siRNA mediated** **TRAFs knockdown on retinoic acid sensitivity in human breast cancer BT549 cells.** (A) TRAF1. (B) TRAF2. (C) TRAF3. (D) TRAF4. (E) TRAF5. (F) TRAF6. (H) TRAF7. All values represent the means of at least three independent experiments ± standard deviation (SD).

**
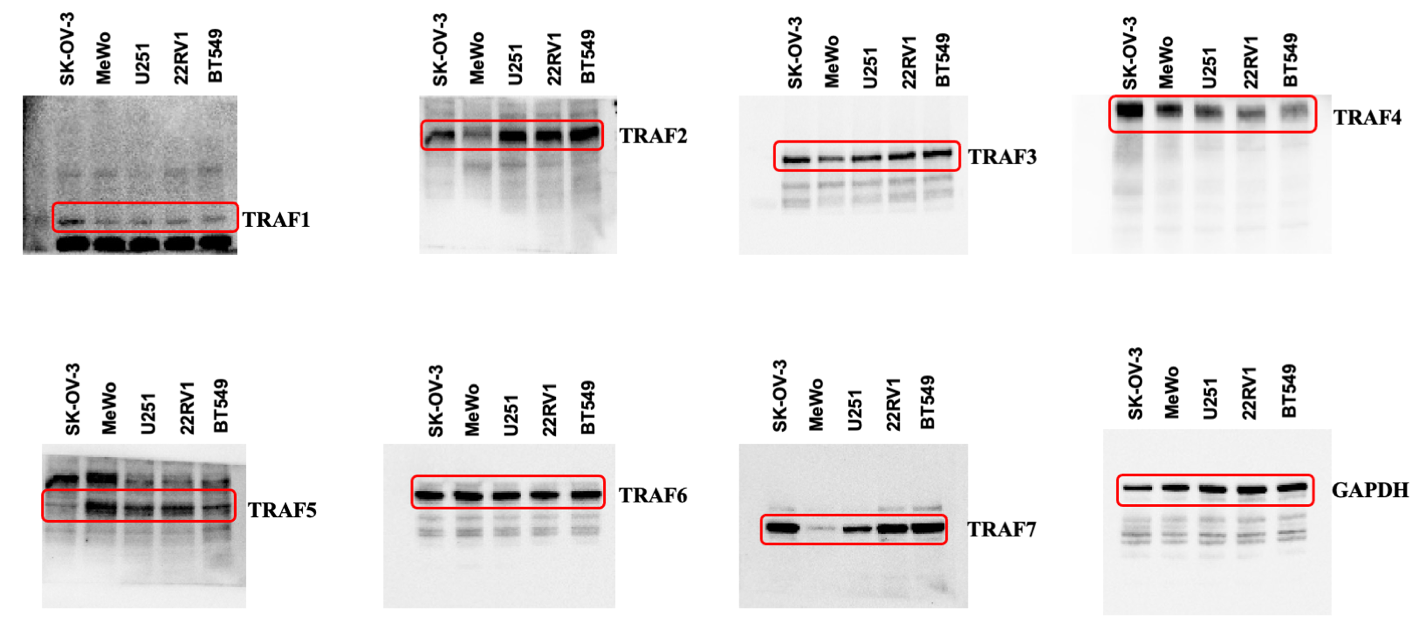
**

**Figure S6. Original pictures of TRAF1/TRAF2/TRAF3/TRAF5/TRAF6/TRAF7 protein blotted in various cancer cell lines (Fig2A of the manuscript)**

**
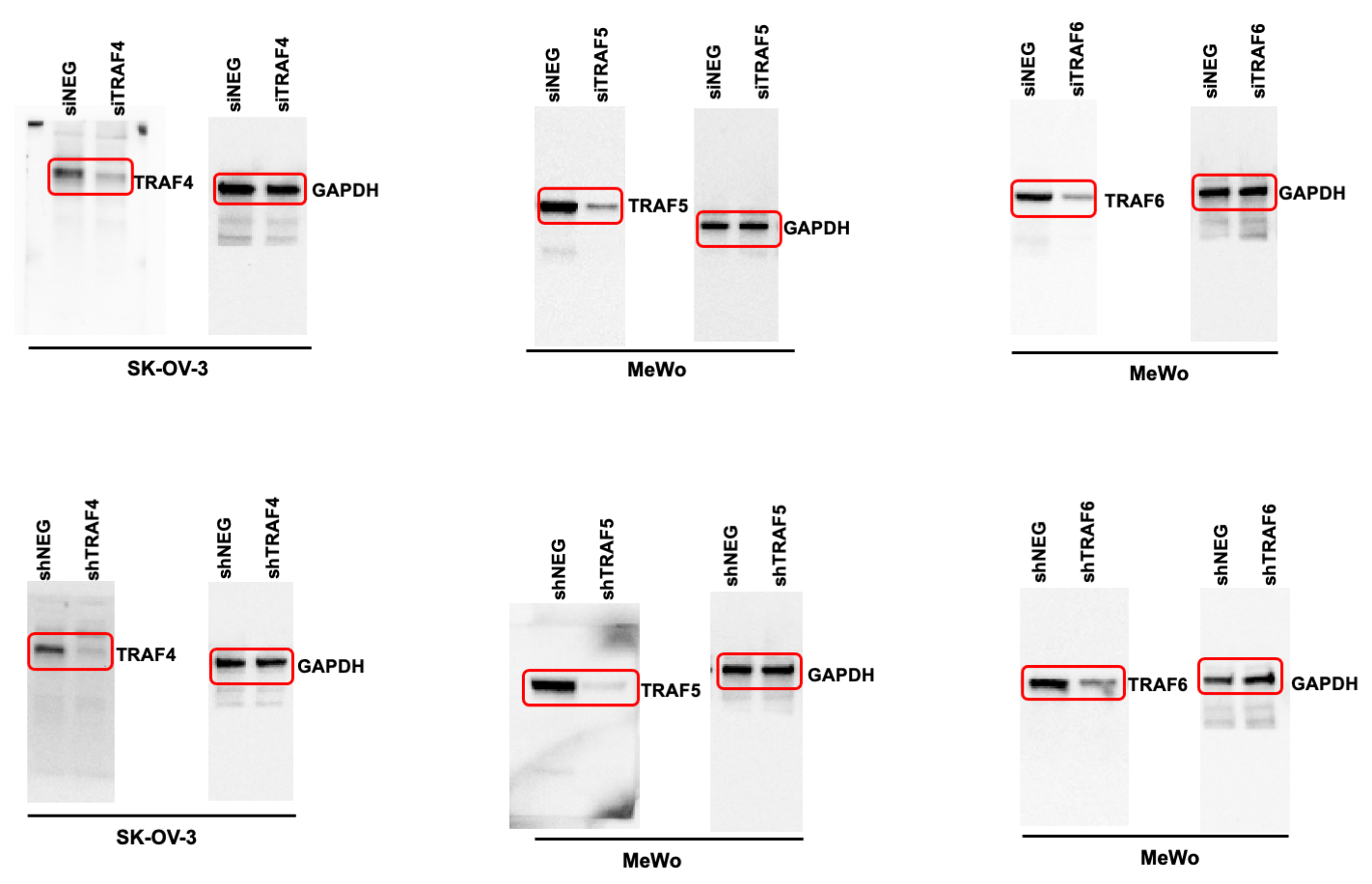
**

**Figure S7. Original pictures of TRAF4/ TRAF5/TRAF6 knockdown (Fig3A- Fig3F of the manuscript)**

**
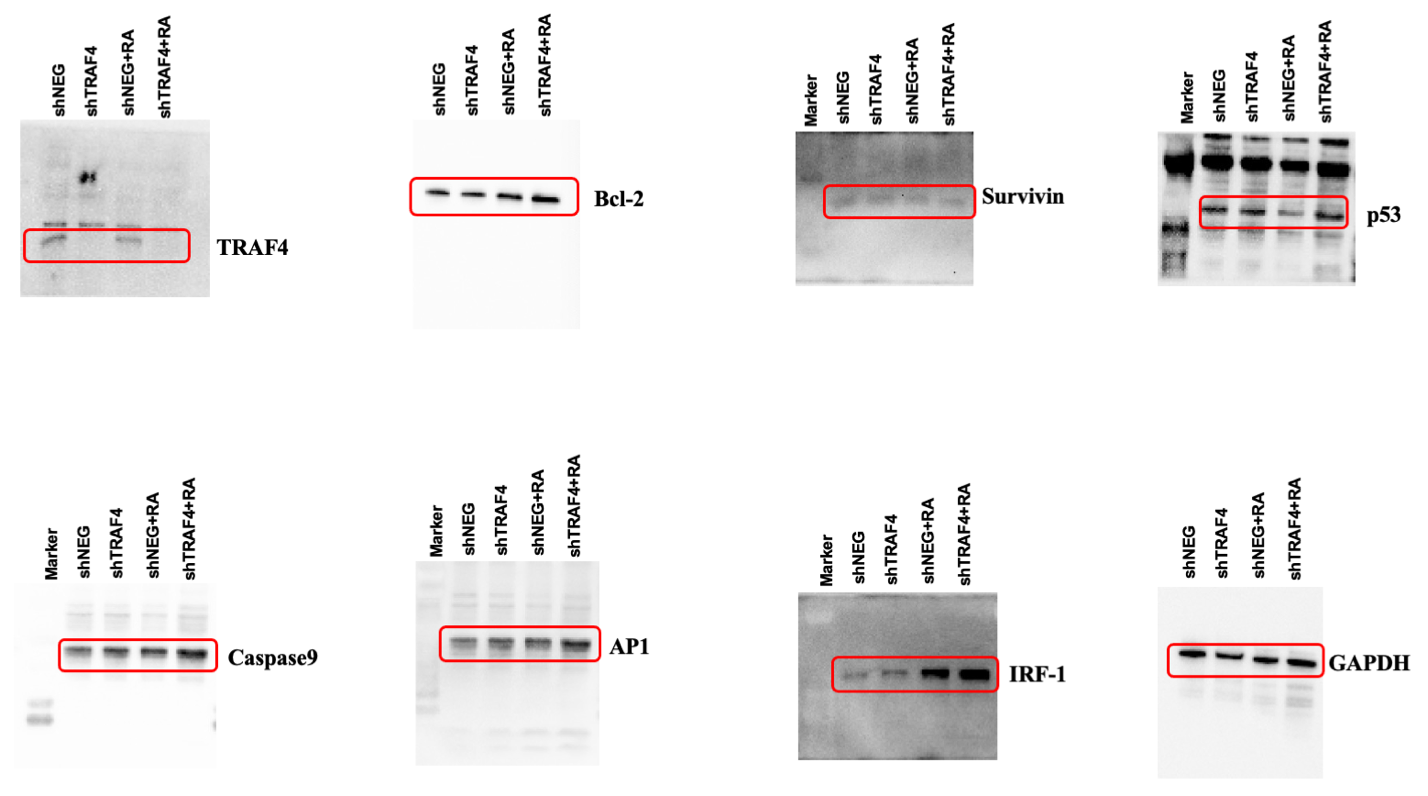
**

**Figure S8. Original pictures of TRAF4 knockdown and apoptosis-related proteins (Fig5B of the manuscript)**

**
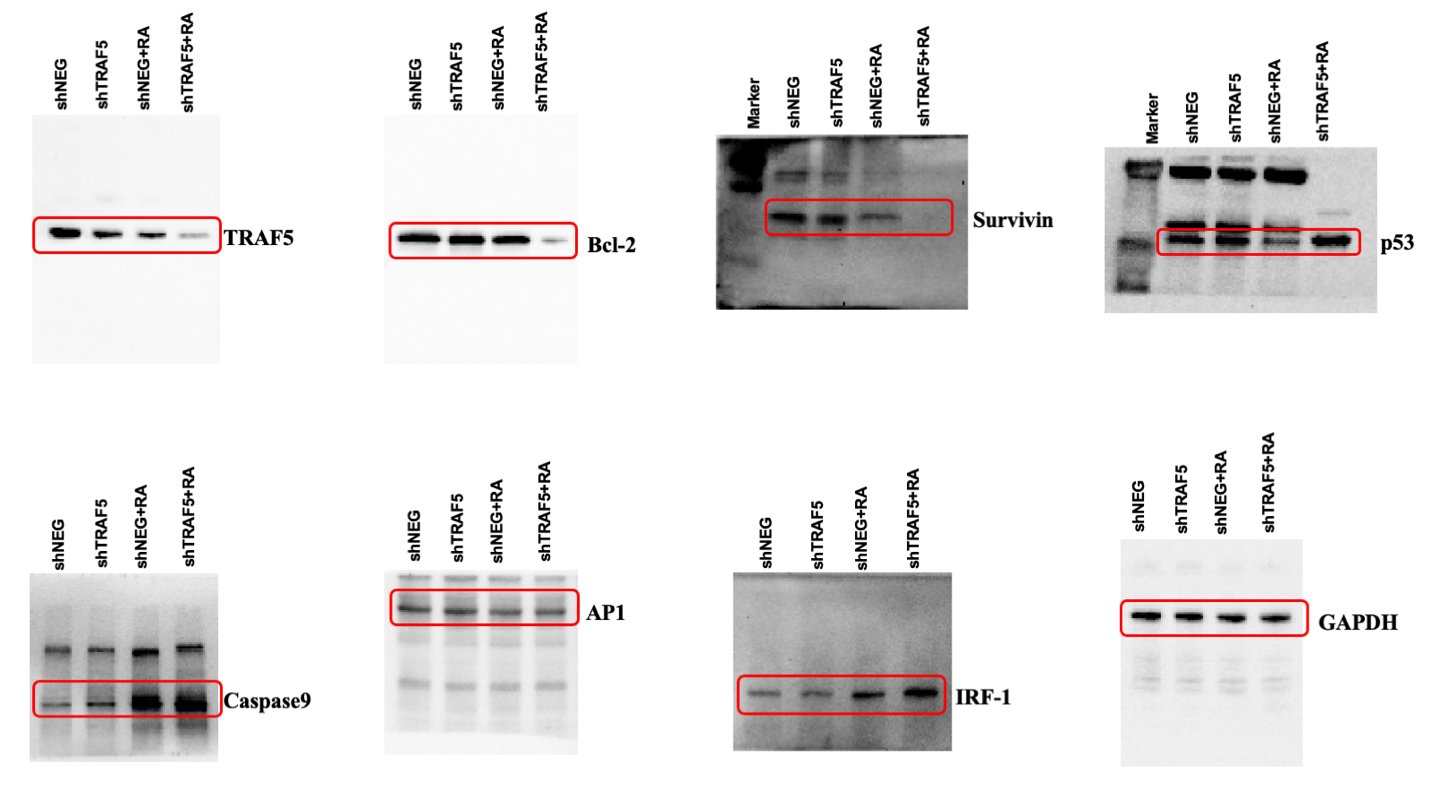
**

**Figure S9. Original pictures of TRAF5 knockdown and apoptosis-related proteins (Fig6B of the manuscript)**

**
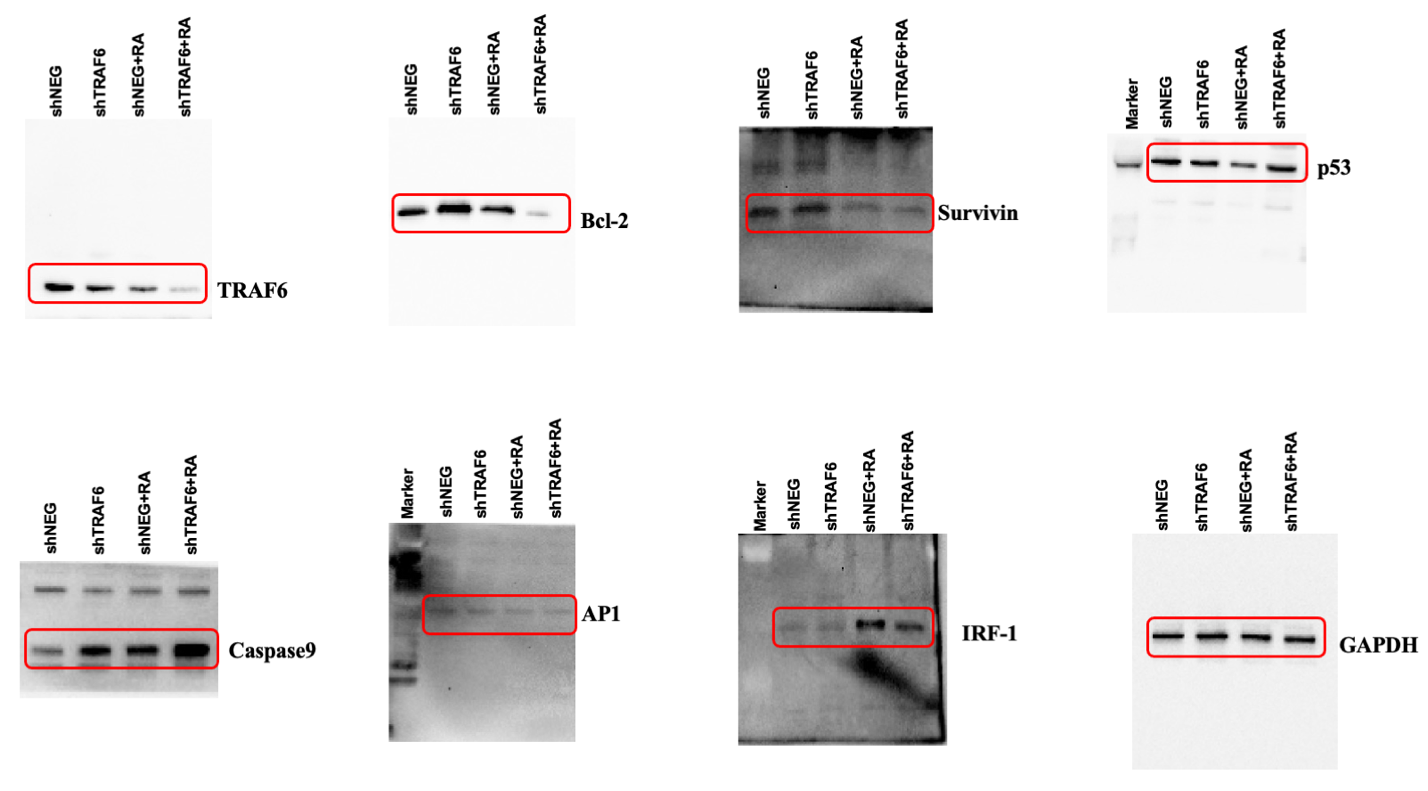
**

**Figure S10. Original pictures of TRAF6 knockdown and apoptosis-related proteins (Fig7B of the manuscript)**
